# Macrophages, as a Promising Strategy to Targeted Treatment for Colorectal Cancer Metastasis in Tumor Immune Microenvironment

**DOI:** 10.3389/fimmu.2021.685978

**Published:** 2021-07-13

**Authors:** Yingru Zhang, Yiyang Zhao, Qi Li, Yan Wang

**Affiliations:** ^1^ Department of Medical Oncology, Shuguang Hospital, Shanghai University of Traditional Chinese Medicine, Shanghai, China; ^2^ Academy of Integrative Medicine, Shanghai University of Traditional Chinese Medicine, Shanghai, China

**Keywords:** colorectal cancer, metastasis, macrophages, targeted treatment, signaling pathways

## Abstract

The tumor immune microenvironment plays a vital role in the metastasis of colorectal cancer. As one of the most important immune cells, macrophages act as phagocytes, patrol the surroundings of tissues, and remove invading pathogens and cell debris to maintain tissue homeostasis. Significantly, macrophages have a characteristic of high plasticity and can be classified into different subtypes according to the different functions, which can undergo reciprocal phenotypic switching induced by different types of molecules and signaling pathways. Macrophages regulate the development and metastatic potential of colorectal cancer by changing the tumor immune microenvironment. In tumor tissues, the tumor-associated macrophages usually play a tumor-promoting role in the tumor immune microenvironment, and they are also associated with poor prognosis. This paper reviews the mechanisms and stimulating factors of macrophages in the process of colorectal cancer metastasis and intends to indicate that targeting macrophages may be a promising strategy in colorectal cancer treatment.

## Introduction

Colorectal cancer (CRC) is the third most common malignancy and the second leading cause of cancer deaths in the world (1). Approximately 50% of CRC patients develop metastatic diseases (2). CRC has a high probability of metastasis following its diagnosis (3, 4), and metastases especially involve the liver (5). Cytotoxic drug chemotherapy is usually the first choice for metastatic CRC (mCRC), but it is accompanied by numerous side effects, and the prognosis is unsatisfactory. Targeted therapy also currently plays a significant role in the treatment of mCRC, while chemotherapy combined with targeted therapy for mCRC is the first-line therapy in clinical treatment (6, 7). Due to the advantages of targeted therapy, such as precision, high efficiency, significantly less toxicity than chemotherapy, and convenient oral administration (8), targeted therapy will become a promising treatment approach. The discovery of novel molecular biomarkers will likely be of great significance for the treatment of mCRC (9, 10). Immune checkpoint inhibitors, including anti-programmed cell death 1 (PD-1), anti-programmed cell death ligand 1 (PD-L1) monoclonal antibodies (MAbs) and CTL-associated antigen 4 (CTLA4) blockade have been confirmed to improve the overall survival rate of patients in different cancer types (11, 12).

The tumor immune microenvironment (TIME) is complex and diverse and mainly includes tumor cells, immune cells, antigens, and cytokines (13). Recently, investigations regarding the TIME have received great interest. Macrophages are one of the most important cells in the TIME. Macrophages promote or inhibit tumor invasion and metastasis through the interaction between various molecules and signaling pathways (14–16). Studies have shown that the high immunoactivity defined by the microsatellite instability (MSI) subtype CRC is associated with the high degree of infiltration of M1 macrophages (17). Therefore, molecular targeted therapy directed at the CRC immune microenvironment is a focal point in the treatment of tumors; targeting macrophages potentially exerts long-term effects for the treatment of mCRC.

## Tumor Immune Microenvironment

The TIME is formed by the interaction between tumor cells, tumor-infiltrating immune cells, epithelial cells, fibroblasts, blood vessels, chemokines, and cytokines (18, 19). In the TIME, adaptive immune effector T cells (20), and innate immunity effector cells, macrophages (21), NK cells (22), and other cells promote the inflammatory response and are involved in antitumor effects. In contrast to antitumor immunity, tumor-associated macrophages (TAMs) (23), and myeloid suppressor cells (MDSCs) (24), and regulatory T cells (Tregs) (25) as immunosuppressive cells also play an immunomodulatory effect in tumor immunity, facilitating the metastasis of tumors. The occurrence and development of tumors are closely associated with antitumor immune cells in the TIME. Conversely, immune cells can also be influenced by products of tumor cells such as cytokines (26) and exosomes (27), and integrate with signaling pathways by activating immune evasion mechanisms, which further induce tumor metastasis.

**Figure 1:**
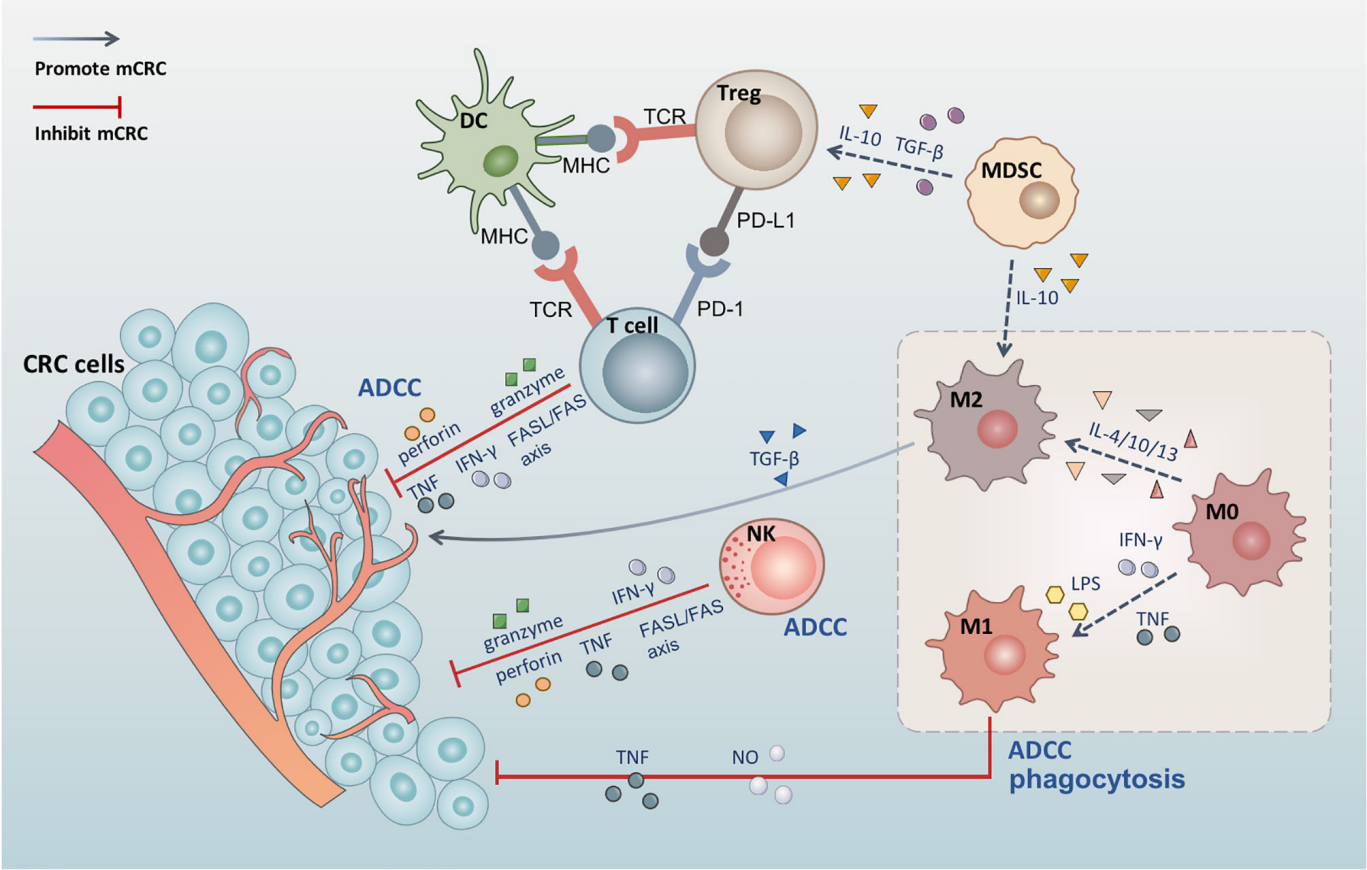
The tumor immune microenvironment of colorectal cancer. DC activates T cells through the combination of the MHC and T cell receptors. Tregs inhibit the immune effects of T cells *via* the PD-1/PD-L1 axis. Both NK cells and T cells can kill tumor cells through the Fas/FasL pathway, the perforin-granzyme pathway, and by releasing TNF and IFN-*γ*. MDSCs can mediate the development of M2 macrophages and Tregs, which depends on IL-10, and produce TGF-*β* to induct Tregs. M0 macrophages polarize into M1 macrophages under the effect of LPS, IFN-*γ*, and TNF, while polarizing into M2 macrophages following induction by IL-4, IL-10, and IL-13. M1 macrophages also induce tumor cell apoptosis through phagocytosis, ADCC, and the release of TNF and NO. M2 secretes TGF-*β* and contributes to angiogenesis, promotes tumor cell EMT, and induces immunosuppressive microenvironment.

In the TIME associated with CRC, various immune cells interact with each other to promote or inhibit the growth, invasion, and metastatic potential of CRC **(** **Figure 1** **)**. Dendritic cells (DCs) activate T cells through the combination of the major histocompatibility complex (MHC) and T cell receptors (28). NK cells can kill tumor cells directly through antibody-dependent cell-mediated cytotoxicity (ADCC) (29), and T cells can kill tumor cells directly through cytotoxicity (30). Both NK cells and T cells can kill tumor cells through the Fas/FasL pathway, the perforin-granzyme pathway (31), and by releasing tumor necrosis factor (TNF) (32). MDSCs can mediate the development of M2 macrophages and Tregs, which depend on IL-10 (33, 34). T cells promote tumor immunity by secreting IFN-*γ* (35), while Tregs inhibit the immune effects of T cells *via* the PD-1/PD-L1 axis (36). Macrophages can promote or inhibit tumor immunity by polarizing into different types and play a critical role in the tumor microenvironment (TME). Furthermore, mutual transformation of macrophages regulates the TIME in CRC.

## The Role of Macrophages in the Metastasis of CRC

Macrophages can differentiate into different subtypes in the TIME and may play dual roles (37). They produce various molecules that interact with other immune cells and tumor cells and further affect the progression in CRC (38). Under the influence of different cytokines and exosomes, three types of macrophages can be identified: naive M0, the M1 subtype with pro-inflammatory effects, and the M2 subtype with immunosuppressive effects (39, 40), which promote or inhibit the progression of CRC.

### Antitumor Effects of Macrophages

The classical activated macrophages are defined by the M1 macrophages whose surface markers are CD86, iNOS, and TNF-α (41–43), M0 macrophages polarize into M1 macrophages under the effect of LPS and IFN-*γ* (44), which are active against pathogen infection and play a significant function in immunity. M1 macrophages can inhibit tumor development by releasing tumor-suppressing molecules, including TNF-α (45). M1 macrophages also induce tumor cell apoptosis through phagocytosis (46), ADCC (47), and release of TNF and nitric oxide (NO) (48). Studies have shown that M1-type macrophages can promote tumor immunity by recruiting cytotoxic T cells (49).

### Tumor-Promoting Effects of Macrophages

Macrophages in the TIME are often called TAMs; M0 macrophages are mostly polarized into M2 macrophages (50) following induction by IL-4, IL-10, and IL-13 (51–53). CD163, CD206 and Arg1 are common surface markers of M2 macrophages (54–56). TAMs are classified as M2 macrophage activation subtype, which can promote the development of CRC in the TIME (57). A research showed that TAMs increased significantly in hepatic metastatic tumors of colorectal cancer (58). TAMs contribute to angiogenesis (59), promote epithelial–mesenchymal transition (EMT) of tumor cells (60), and activate immunosuppression (61), promoting the metastasis of CRC. Comfortingly there is evidence that macrophages have plasticity (62, 63), and various stimuli, including drugs or M1 exosomes, can cause macrophages to alter their phenotype and reprogram M2 toward M1, inhibiting tumor development (64, 65). Therefore, targeting macrophages will be a potential strategy for the treatment of colorectal cancer metastasis.

## Signaling Pathways Involving Macrophages in CRC Metastasis

**Figure 2:**
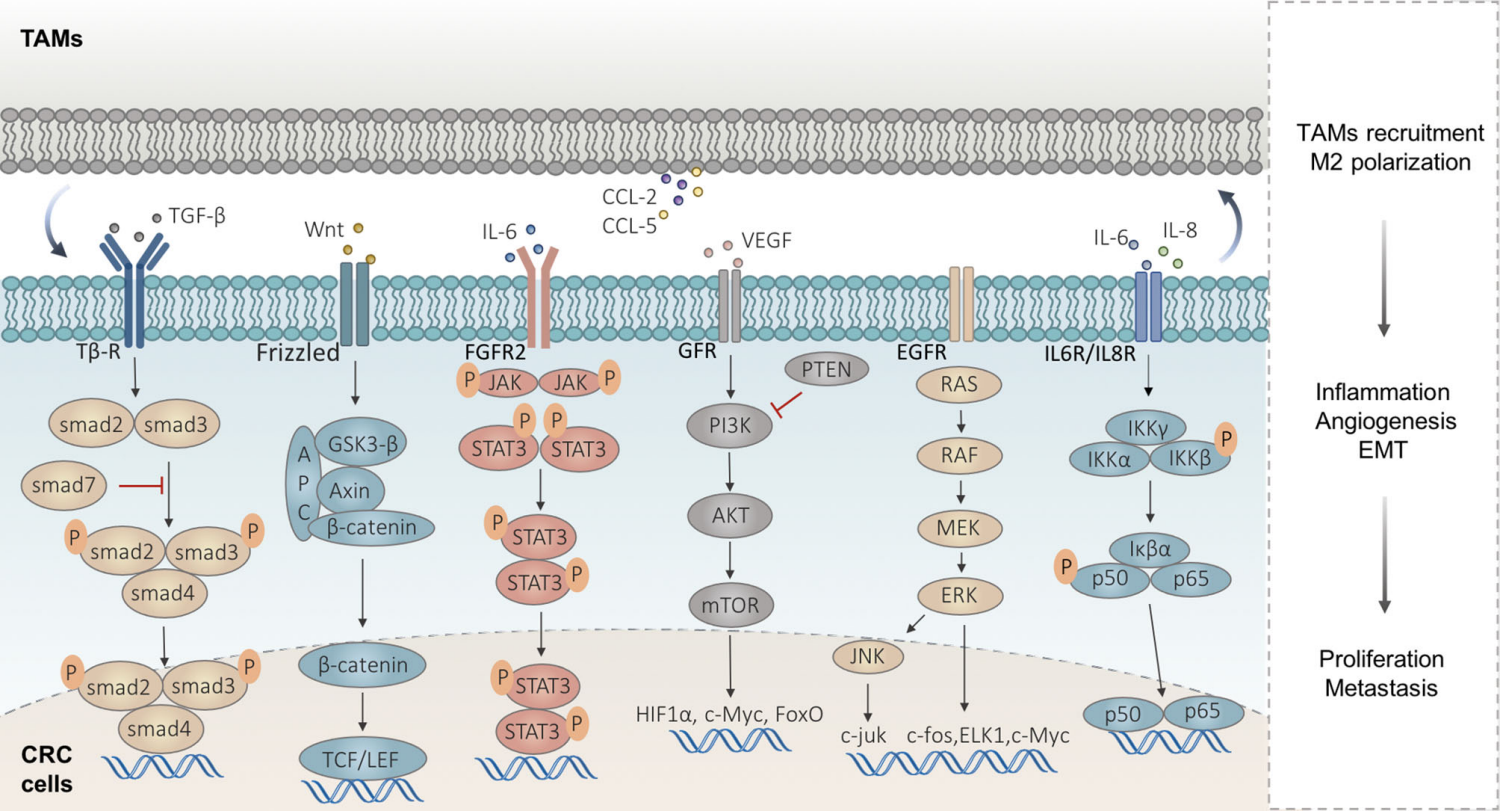
Signaling pathways implicated in macrophages in CRC metastasis. Wnt/*β*-catenin signaling is activated in highly proliferative CRC accompanied by the TAM infiltrates. TAMs upregulate the VEGF-A of CRC cells and activate the NF-*κ*B signaling pathway by secreting IL-6 and IL-8. TAMs produce IL-6 to activate the JAK/STAT3 signaling in CRC cells and lead to the EMT, which in turn lead to the CCL2/5 secreted by CRC cells, to promote macrophage recruitment. Some exosomes carrying miRNAs derived from CRC cells regulate PTEN, by activating the PI3K/Akt signaling to induce M2-like TAM polarization, and in turn, TAMs promote CRC metastasis by enhancing EMT and secreting VEGF. The abnormal activation of the MAPK signaling pathway can induce tumor cell proliferation and participates in the development and metastasis of CRC. TAMs facilitate the EMT program involved in the CRC metastatic process *via* TGF-*β*/Smad signaling.

The immune cells in the TIME regulating CRC development are achieved by the secreted immune molecules and CRC cell-surface receptors, activating the intracellular signaling pathways involving macrophages, including Wnt/*β*-catenin, NF-*κ*B, PI3K/AKT, JAK/STAT3, MAPK, and TGF-*β*/Smad signaling pathways **(** **Figure 2** **)**.

### Wnt/*β*-Catenin Signaling Pathway

The wnt/*β*-catenin signaling pathway promotes the development of CRC by regulating the growth, differentiation, and migration of tumor cells (66). Abnormalities of the wnt signaling, such as the APC gene mutation, are common in CRC, which can promote the development of CRC (67). A global transcriptome immune classification experiment for CRC solid tumors showed that most patients had APC mutations (68). Activation of the wnt signaling pathway leads to granulocyte recruitment and tumor invasion, and abnormal wnt signaling directly alters the antineoplastic activities of effector T cells, helper T cells, and Tregs, suppressing tumor immunity (69). In highly proliferative colorectal tumors, wnt/*β*-catenin signaling is activated and abundant *β*-catenin accumulates in the nucleus, accompanying the immune cell infiltrates including TAMs (70).

### NF-*κ*B Signaling Pathway

Nuclear factor-*κ*B (NF-*κ*B) is a critical molecule underlying the relationship between inflammation and tumor immunity and is involved in the growth and development of CRC (71). Various extracellular factors including proinflammatory cytokines, LPS, and growth factors lead to I*κ*B protein phosphorylation and ubiquitination and then degradation, freeing NF-*κ*B/Rel complexes and transferring into the nucleus for transcription, promoting EMT, angiogenesis, and metastasis (72, 73).

Transcription factors mediated by NF-*κ*B are associated with MDSC activation (74). NF-*κ*B expression is upregulated in CD4+TIM-3+ tumor-infiltrating lymphocytes, inducing the expression of inflammatory factors and T cell failure, which in turn further facilitates the metastasis of CRC (75). The NF-*к*B pathway is activated in P2X7R overexpressed CRC cells, cytokines increasing leads to the recruitment of TAMs (76). TAMs can significantly upregulate the vascular endothelial growth factor-A (VEGF-A) of CRC cells and activate the NF-*κ*B signaling pathway by secreting IL-6 and IL-8, promoting CRC metastasis (77). In patients with CRC, high levels of p50-NF-*κ*B + TAMs at the invasive margin are associated with poor prognosis following surgical intervention for excision of tumors. The p50-NF-*κ*B + TAMs participate in the development of CRC by reducing recruitment and antitumor activity of T cells, which confirms that the NF-*κ*B pathway is a significant signaling pathway promoting CRC metastasis (78). However, the role of NF-*κ*B in colorectal cancer remains controversial. A research found that for macrophages, the activation of NF-*κ*B can promote M1-polarization, so as to play an antitumor effect (79).

### JAK/STAT3 Signaling Pathway

In the TIME, the abnormal activation of the JAK/STAT3 signaling contributes to an immunosuppressive tumor microenvironment, which promotes tumor growth and metastasis (80). Studies have indicated that TAMs produce pro-inflammatory cytokine IL-6 to activate the JAK/STAT3 signaling in CRC cells and lead to epithelial–mesenchymal transition (EMT) involved in tumor progression (81), which in turn leads to the CCL2 secreted by CRC cells, to promote macrophage recruitment, while, inhibition of CCL2 or IL6 can break this crosstalk (82).

On the other hand, IL-6 can upregulate other inflammatory factors such as CCL2 and CCL5, and then recruit macrophages (83, 84). Furtherly, the recruited macrophages in turn secret IL-6 to activate JAK2/STAT3 signaling, promoting tumor metastasis (85). CCL2 significantly increases the number of MDSCs and M2-like TAMs mediated by STAT3, suppresses T cells, and promotes immune evasion in CRC (86). All the above studies have shown that CRC cells and TAMs influence each other to promote tumor development.

### Phosphatidylinositol 3 Kinase/AKT Signaling Pathway

The phosphatidylinositol 3 kinase (PI3K)/AKT signaling pathway is one of the most activated pathways in human cancer (87, 88), which can regulate the immunosuppressive microenvironment, promoting immune cell exhaustion and inhibiting antitumor activity (89). PD-1/PD-L1 blockade can rescue depleted CD8+ T cells *via* the PI3K/Akt/mTOR signaling pathway (90). Tumor cells overexpressing T cell immunoglobulin mucin-4 (TIM-4) activate PI3K/AKT/mTOR signal transduction and recruit TAMs, promoting proliferation and tumor matrix remodeling in CRC (91). Some exosomes carrying miRNAs (miR-25-3p, miR-130b-3p, miR-425-5p) derived from CRC cells regulate PTEN by activating PI3K/Akt signaling to induce M2-like TAM polarization, and in turn, TAMs promote CRC metastasis by enhancing EMT and secreting vascular endothelial growth factor (VEGF) (92). In addition, inhibition of AKT can effectively limit the differentiation of T cells and enhance antitumor effects *in vivo* (93).

### MAPK Signaling Pathway

The mitogen-activated protein kinase (MAPK) signaling pathway is one of the most important bridges for converting extracellular signals to intracellular responses (94). As the key pathway participating in cell proliferation, the abnormal activation of the MAPK signaling pathway can induce tumor cell proliferation and participate in the development and metastasis of CRC (95, 96). The homeoprotein Six1 is associated with poor prognosis in CRC (97). Six1 overexpression promotes CRC growth and metastasis by stimulating angiogenesis and recruiting TAMs, accompanied by MAPK activation in CRC cells (98). In addition, a study demonstrated that TAMs interact with CRC cells inducing EMT in CRC cells by activating the MAPK pathway in TAMs, and then promoting the metastasis of CRC (77). The crosstalk in TAMs and CRC cells reveals the significant role of TAMs in the development of CRC, which provides a powerful argument for targeting TAMs in CRC treatment.

### TGF-*β*/Smad Signaling Pathway

In tumor stroma, M2-polarized TAMs secrete transforming growth factor-beta (TGF-*β*) *via* the miR-34a/VEGF axis and promote invasion and metastasis of CRC (99). There are also some studies that found that *in vitro*, cytokines induced M2 macrophages to produce TGF-*β*1 *via* the VEGF/VEGFR2 signaling pathway (57). TAMs facilitate the EMT program involved in the CRC metastatic process *via* TGF-*β*/Smad2,3–4/Snail signaling (100, 101). A multicolor histology analysis indicated that patients with poor clinical outcomes may also have infiltration of T cells in tumor tissues, but always with high TGF-*β* expression and high TAM density (102), which reveals the critical role of TAMs in CRC metastasis.

Generally, in the immune microenvironment, TGF-*β* and IL-6 are required for the development of Th-17 cells which produce IL-17 (103). The production of IL-17 is positively correlated with distant colon tumorigenesis (104). However, different from the local tumor immune response, studies have found that in non-tumor tissues of cancer patients, the increase of Th-17 cells and IL-17 can enhance M1 polarization while inhibiting M2 polarization (105), which indicates the complex role of TGF*β* in human immunity.

## Molecules Related to Macrophages in CRC Metastasis

Molecular targeted therapy is a promising method for CRC therapy, especially in mCRC. Macrophages play a pivotal role in the metastatic niche, and many molecules, including IL-4, IL-10, and IL-13, can promote macrophages to polarize into M2. In turn, M2 can secrete IL-6, IL-8, and other inflammatory factors to promote the proliferation and metastasis of cancer cells. Therefore, macrophages act as important mediators of the development of CRC. Targeting macrophages may provide a new strategy for the treatment of CRC. All molecules related to targeting macrophages are listed in **Table 1**.

**Table 1:** Molecules related to TAMs in CRC metastasis.

Molecules	Types	Expression in mCRC	Mechanism	Effects in CRC	References
**lncRNA RPPH1**	Non-coding RNA	Up	Mediates the polarization of M2	Promote	(106, 107)
**lncRNA HLA-F-AS1**	Non-coding RNA	Up	Mediates the polarization of M2	Promote	(108)
**miR-21-5p**	Non-coding RNA	Up	Derived by M2-macrophages, downregulates the expression of BRG1	Promote	(109)
**miR-25-3p**	Non-coding RNA	Up	Activates CXCL12/CXCR4 axis, induces M2 polarization	Promote	(92)
**miR-130b-3p**	Non-coding RNA	Up	Activates CXCL12/CXCR4 axis, induces M2 polarization	Promote	(92)
**miR-155-5p**	Non-coding RNA	Up	Derived by M2-macrophages, downregulates the expression of BRG1	Promote	(109)
**miR-425-5p**	Non-coding RNA	Up	Activates CXCL12/CXCR4 axis, induces M2 polarization	Promote	(92)
**miR-934**	Non-coding RNA	Up	Promotes CRC liver metastasis by regulating the interaction between CRC cells and TAMs	Promote	(110)
**sST2**	Cytokine receptor	Down	Suppresses IL-33-induced angiogenesis, macrophage infiltration and polarization	Inhibit	(111)
**CCL2**	Cytokines	Up	Promotes the recruitment of macrophages	Promote	(82, 86, 98, 112)
**CCL5**	Cytokines	Up	Promotes the recruitment of macrophages	Promote	(83, 113)
**CCL17**	Cytokines	Up	Upregulates in M2-like TAMs, induces an immunosuppressive environment	Promote	(111)
**CXCL1**	Cytokines	Up	Secreted by TAMs, forms a pre-metastatic niche, promotes liver metastasis	Promote	(114)
**TGF-β**	Cytokines	Up	Secreted by TAMs, facilitates EMT in CRC	Promote	(101)
**VEGF**	Cytokines	Up	Augments the recruitment of TAMs	Promote	(98, 99, 115, 116)
**CSF-1**	Cytokines	Up	Augments the recruitment of TAMs	Promote	(98)
**IL-1β**	Cytokines	Up	Regulates the crosstalk between TAMs and CRC cells	Promote	(115, 116)
**IL-6**	Cytokines	Up	Upregulates CCL2 and CCL5, and then recruits TAMs	Promote	(81)
**IL-10**	Cytokines	Up	Induces the M2 polarization	Promote	(117)
**LUM**	Metabolites	Up	Regulates macrophage polarization	Promote	(118)
**ABHD5**	Metabolites	Down	Low-level expressed in migratory TAMs, upregulates the MMPs	Promote	(119)
**PRL-3**	Phosphatases	Up	Activates the MAPK pathway in TAMs to promote EMT	Promote	(77, 120, 121)
**Shp2**	Phosphatases	Up	Promotes the maturation of TAMs	Promote	(122)
**KRS**	proteases	Up	Induces M2 polarization of macrophages	Promote	(122)
**CTSK**	proteases	Up	Induces M2 polarization of macrophages	Promote	(123)
**Gas6**	protein	Up	Induces M2 polarization of macrophages	Promote	(122)
**NLRC4**	Inflammasome	Up	Regulates the crosstalk between TAMs and CRC cells	Promote	(116)
**NLRP3**	Inflammasome	Up	Regulates the crosstalk between TAMs and CRC cells	Promote	(115)
**Wnt5a**	Secreted protein	Up	Activates macrophages polarization	Promote	(112, 117)
**S100A8**	Calcium- and zinc-binding protein	Up	Activates the NF-*κ*B pathway in macrophages	Promote	(124)
**GRP78**	Glucose regulated protein	Up	Upregulates by TAMs, promotes STAT3 phosphorylation	Promote	(125)
**P2X7R**	Purine receptor	Up	Leads to the recruitment of TAMs *via* NF-*κ*B pathway	Promote	(76, 126)
**LAYN**	Hyaluronan receptor	Up	Activates macrophages polarization and associates with poor prognosis of patients	Promote	(127)
**COX-2**	Cyclooxygenase	Up	Promotes the differentiation of M2 macrophages and reduces the expansion of M1 macrophages	Promote	(128)
**PGE2**	Prostaglandin E2	Up	Promotes the differentiation of M2 macrophages and reduces the expansion of M1 macrophages	Promote	(128, 129)
**IDO**	Indoleamine 2,3-dioxygenase 1	Up	Promotes Tregs and M2 macrophages cooperative effects, leads to immunosuppression	Promote	(130)
**KYN**	Kynurenine	Up	Promotes Tregs and M2 macrophages cooperative effects, leads to immunosuppression *via* AHR axis	Promote	(130)

### Non-Coding RNA

Studies have demonstrated that non-coding RNA molecules play a pivotal role in the polarization process of TAMs. The overexpression of the long non-coding RNA (LncRNA) RPPH1 has been associated with advanced tumor-node-metastasis (TNM) stages and poor prognosis (106). Exosomes derived from CRC cells transport RPPH1 into macrophages and mediate M2-like polarization to promote CRC cell proliferation and metastasis (107). The lncRNA HLA-F-AS1 regulates the expression of profilin 1(PFN1) in CRC-derived EVs by inhibiting miR-375, and then, in turn, mediates the M2 phenotype polarization of macrophages (108), promoting the CRC metastasis.

Several miRNAs including miR-25-3p, miR-425-5p, and miR-130b-3p induce macrophage M2 polarization by activating the CXCL12/CXCR4 axis in CRC metastasis (92). The tumor-derived exocrine miR-934 can promote CRC liver metastasis by regulating the interaction between CRC cells and TAMs (110). In addition, exosomes carrying miRNA-106b-5p promote the M2-like polarization of macrophages and induce the EMT in CRC cells, which is implicated in the crosstalk between tumor cells and TAMs (131). Brahma-related gene-1 (BRG1) is the core subunit of switch/sucrose nonfermentable (SWI/SNF) family complexes (132). M2-macrophages-derived exosomes carry miR-155-5p and miR-21-5p to CRC cells and combine with the BRG1 coding sequence to downregulate the expression of BRG1, promoting the metastasis of CRC (109).

Thus, we can achieve the effect of blocking M2-like polarization or of blocking tumor from promoting secretion from TAMs by inhibiting the above non-coding RNA, thereby controlling the tumor-promoting effect of macrophages in the tumor microenvironment of CRC.

### Cytokines

Many cytokines are involved in the polarization of TAMs, some of them active in downstream signaling pathways to promote CRC metastasis. Studies have revealed that complex chemokine networks can affect cancer progression *via* the recruitment and activation of TAMs. The increased expression of CCL17 in DCs and M2-like TAMs in tumors induces an immunosuppressive environment; CCL17 expression has been used as a marker for M2-like immunosuppressive macrophage polarization (133). CCL5, secreted by TAMs, inhibits T-cell-mediated killing of CRC cells and promotes immune escape by stabilizing PD-L1 (113). In addition, CRC cells secrete VEGF-A and then stimulate TAMs to produce CXCL1 in primary tumors. The increased release of CXCL1 transfer to the liver *via* blood circulation recruits CXCR2-expressing MDSCs to form a pre-metastatic niche, promoting liver metastasis (114).

In addition, sST2, a soluble isoform of the IL-33 receptor (ST2), suppresses angiogenesis, macrophage infiltration, and macrophage M2 polarization induced by IL-33 (111). M2-polarized TAMs secrete TGF-*β* (100), which regulates the miR-34a/VEGF axis to facilitate CRC cell proliferation and invasion (99). Wnt5a is highly expressed in TAMs and can induce M2 polarization by regulating the secretion of IL-10, which is mediated by the CaMKII-ERK1/2-STAT3 pathway (117). Furthermore, Wnt5a^+^TAMs promote CRC development which also depends on CCL2 secretion mediated by the CaMKII-ERK pathway (112).

Therefore, we can regulate the immunosuppressive microenvironment by inhibiting the cytokine-induced macrophage M2-like polarization, decrease the recruitment of immunosuppressive cells, and enhance tumor immunity for CRC metastasis.

### Macrophage-Related Metabolites

Many metabolites are also related to the metastasis of CRC. These factors are involved in tumor progression and offer a new direction for mCRC treatment. A small leucine-rich proteoglycan lumican (LUM) regulates macrophage polarization in colorectal adenocarcinoma and induces immune escape in the microenvironment of CRC (118). TAMs play an important role in tumor invasion and can migrate with tumor cells during the process of tumor metastasis. TAMs exhibit a heterogeneous expression of the hydrolase domain containing the triglyceride hydrolytic activator 5 (ABHD5) which is expressed in low-level in migratory TAMs and upregulate matrix metalloproteinases (MMPs) involved in CRC metastasis (119). According to the above, we consider that the regulation of Lum or ABHD5 can target TAMs to prevent the metastasis of CRC.

### Phosphatases and Proteases

During the metastasis of CRC, phosphatases and proteases play an important role. It has been found that the protein tyrosine phosphatase-3 (PRL-3) (120) increases CCL26 secretion to stimulate TAM infiltration (121) and activates the MAPK pathway in TAMs, ultimately initiating EMT. On the other hand, PRL-3 can directly induce angiogenesis *via* NF-*κ*B signaling (77). Tyrosine phosphatase 2 (Shp2), which contains two homologous domains of Src, is a non-receptor tyrosine phosphatase encoded by the PTPN11 gene and is positively correlated with tumor metastasis. Studies have shown that Shp2 promotes the maturation of TAMs by activating RAS, and it is associated with PD-1 signaling in T cells (134). KRAS-positive CRC cells secrete cytokines, including growth arrest-specific 6 (Gas6) and cause M2 macrophages polarization and infiltration. In addition, CAFs are activated by communication between CRC cells and TAMs, which remodels the environment of CRC metastasis for cancer cell dissemination (122, 135). Cathepsin K (CTSK) is a lysosomal cysteine protease, which is implicated in signal transduction in cancer cells. CTSK, secreted by CRC cells, induces the polarization of M2 macrophages and mediates the interaction between the gut microbiota imbalance and CRC metastasis, and CTSK overexpression in CRC predicts advanced progression and poor prognosis (123). Thus, it can be seen that the downregulation of these enzymes can block the interaction between TAMs and CRC cells and then inhibit the development of CRC.

### Other Biomolecules

Many other biomolecules are also involved in TAM-mediated CRC metastasis. Both nucleotide-binding oligomerization domain (NOD)-like receptor C4 (NLRC4) and NOD-like receptor family pyrin domain containing 3 (NLRP3) are the main components of the inflammasome, which can increase TAM infiltration and IL-1β production, and promote CRC metastasis by regulating the crosstalk between TAMs and CRC cells (115, 116).

The P2X purine receptor 7 (P2X7R) expressed in tumors leads to the recruitment of TAMs *via* the NF-*κ*B pathway, which facilitates the angiogenesis and the progression of CRC (76, 126). Tumor cells characterized by the overexpression of homologous protein Six1 can raise the recruitment of TAMs by increasing the expression of CSF-1, CCL2, CCL5, and VEGF, promoting CRC metastasis (98). S100 calcium-binding protein A8 (S100A8) can activate NF-*κ*B signaling in macrophages and upregulate IL-1β and TNF-α in TME and augment the migration of CRC cells (124). LAYN, a cell surface hyaluronan (HA) receptor, may be used as a prognostic biomarker for CRC, and it is associated with immune infiltration including TAMs (127). Complement 5a expressed in CRC cells activates macrophage polarization, which in turn facilitates CRC liver metastasis *via* the NF-*κ*B pathway (136).

M2-like macrophages have been reported to upregulate the expression of the glucose-regulated protein of 78 kDa (GRP78) in tumor cells, promoting STAT3 phosphorylation, leading to the downstream inflammatory factors including IL-1β and TNF-α upregulation, which facilitates tumor progression (137).

COX-1 and COX-2 are two isozymes of cyclooxygenase (COX). COX-2 has been found in high levels in CRC (125). Studies have confirmed that COX-2 is a promoting factor for liver metastasis of CRC, and it can convert arachidonic acid into prostaglandin E2 (PGE2) (138, 139). TAMs are the main source of COX-2 in intestinal tumors; PGE2-bound EP4 promotes the differentiation of immunosuppressive M2 macrophages and reduces the expansion of immunostimulatory M1 macrophages (128). PGE2 also enhances the tumor infiltration of M2 macrophages and promotes the development and metastasis of CRC (129).

In addition, Indoleamine 2,3-dioxygenase 1 (IDO) suppresses T cell immunity by catabolizing tryptophan into kynurenine (KYN) and promotes CD8+ T cell exhaustion (140). In IDO-expressing tumors, Tregs cooperate with M2-like macrophages, promoting immune suppression *via* Kyn-aryl hydrocarbon receptor (AHR) axis (130). Studies have shown that in tumor tissues, the levels of IDO1 and its catabolite KYN are higher in late stages (stages III and IV) than in early stages (stages I and II) of CRC patients (141). Also, IDO was found to be negatively correlated with the survival rate of patients (142).

From the above data, we infer that by interfering with these molecules in TIME, we can directly or indirectly block the crosstalk between TAMs and CRC cells and then inhibit the progression of the tumor.

## Clinical Development of Targeted Therapy in CRC Metastasis

In recent years, the development and applications of clinical targeted drugs have been increasing. In the treatment of CRC metastasis, targeted drugs that have entered clinical application stage or clinical trials include bevacizumab, ramucirumab, cetuximab, panitumumab, trastuzumab, regorafenib, lapatinib, erlotinib, napabucasin, sym004, and pimasertib **(** **Table 2** **)**.

**Table 2:** Targeted drugs of the treatment in mCRC.

Targeted drugs	Types	Target	Mechanism	Association with macrophage	References
**Bevacizumab**	Human monoclonal IgG1 antibody	VEGF	Inhibits angiogenesis of tumor	Inhibits the infiltration of TAMs	(143–146)
**Ramucirumab**	Human monoclonal IgG1 antibody	VEGF	Inhibits angiogenesis of tumor	Inhibits the infiltration of TAMs	(144, 145)
**Cetuximab**	Human monoclonal IgG1 antibody	EGFR	Inhibits angiogenesis and vascular endothelial permeability	Repolarizes TAMs from M2-like to M1-like phenotypes	(147–149)
**Panitumumab**	Human monoclonal IgG2 antibody	EGFR	Inhibits angiogenesis and vascular endothelial permeability	Recruits myeloid effector cells such as M1 macrophages and PMN for tumor cell killing by ADCC	(47, 148)
**Trastuzumab**	Human monoclonal IgG antibody	HER2	Blocks the growth of cancer cells	Increases macrophage levels and phagocytosis	(146, 150)
**Lapatinib**	Human monoclonal IgG antibody	HER2	Blocks the growth of cancer cells	Reduces the content of TAMs in TIME	(150, 151)
**Regorafenib**	Multi-kinase inhibitor	VEGF	Inhibits angiogenesis of tumor	Reduces the content of TAMs, increase M1 polarization of macrophages	(152–154)
**Erlotinib**	EGFR tyrosine kinase inhibitor	EGFR	Blocks tumor growth by inhibiting the activity of tyrosine kinase	Reduces the content of TAMs, increases M1 polarization of macrophages	(155–157)
**Napabucasin**	Inhibitor of STAT3	STAT3	Inhibits tumor metastasis and recurrence	Reduces the polarization and infiltration of M2	(158, 159)
**Sym004**	Anti-EGFR Antibody Mixture	EGFR	Inhibits tumor growth and metastasis	Reduces the polarization and infiltration of M2	(160)
**Pimasertib**	MEK inhibitor	MAPK	Inhibits the development and metastasis of CRC	Reduces the polarization and infiltration of M2	(161)

TAMs are one of the causes of tumor angiogenesis and tumor immune escape mechanisms, and targeted treatment of macrophages represents a new challenge and may become a novel strategy for cancer therapy. In the TME, the antiangiogenic drugs bevacizumab and ramucirumab can bind to human VEGF and block its biological activity (143–145); cetuximab and panitumumab bind to the epidermal growth factor receptor (EGFR), repolarize TAMs from M2-like to M1-like phenotypes, recruit myeloid effector cells such as M1 macrophages and PMN for tumor cell killing by ADCC (147–149), and inhibit angiogenesis and vascular endothelial permeability (162–164), and thus block M2 cell infiltration in the inflammatory environment and impede tumor development (165–167). HER2 is positively expressed in CRC, and some studies have shown that trastuzumab and lapatinib, drugs targeting HER2, can inhibit tumor formation by increasing macrophage levels and phagocytosis, and by increasing the infiltration of immune cells, it exerts a therapeutic effect on CRC metastasis (83, 146, 150, 151).

Besides the mentioned monoclonal antibodies above, there are several targeted drugs proposed for the treatment of mCRC. Regorafenib, a multi-kinase inhibitor, not only plays an anti-angiogenesis role by inhibiting VEGF but also induces M2 to M1 TAM polarization (152–154). Erlotinib can inhibit the phosphorylation of intracellular tyrosine kinases associated with EGFR, reducing the content of TAMs (155–157). Napabucasin inhibits the STAT3, which is associated with tumor stemness. Due to the increasing evidence supporting the overexpression of STAT3 in CRC cells, it can be inferred that napabucasin may reduce the STAT3-mediated TAM infiltration and chemoresistance (158, 159). Furthermore, as previously mentioned, in the TIME, the activation of the MAPK pathway in CRC cells can promote the recruitment of TAMs. Pimasertib, a drug targeting MAPK, has also been shown to be effective in phase I clinical treatment of mCRC (161). Sym004, a dual-antibody mixture targeting non-overlapping EGFR epitopes, can inhibit the infiltration of macrophages in TIME, thus providing a good therapeutic approach for mCRC (160, 166). All the above-mentioned pre-clinical and clinical-stage drugs are implicated in macrophage, which suggests that the development of macrophage-targeted drugs have long-term clinical significance.

## Clinical Trial Drugs Targeting Tams in Metastatic CRC

There are drugs targeting macrophages to treat mCRC in clinical trials, either as a single therapy or in combination with chemotherapy or immunotherapy (**Table 3**). Studies have shown that the expression of PD-1 by TAMS can inhibit the phagocytosis of macrophages against tumors and tumor immunity (173). Also, macrophage colony-stimulating factor 1 (CSF-1) plays an important role in macrophage differentiation and angiogenesis (174). In the clinical research on the treatment of mCRC, there are many studies on the anti-CSF-1 receptor (CSF-1R) and anti-PD-1/PD-L1 targeted drugs. RG7155 (emactuzumab) is a humanized mAb that binds to CSF1R and blocks its dimerization. In mouse models of CRC, RG7155 treatment reduces the infiltrated TAMs and increase CD8(+)/CD4(+) T cell ratio (168). Pexidartinib and Durvalumab are anti-CSF1R and anti-PD-L1 drugs respectively. Recently, clinical studies are evaluating the safety and activity of their combination in patients with advanced/metastatic CRC and clinically active pancreatic cancer (169).

**Table 3:** Current clinical trial drugs targeting TAMs in mCRC treatment.

Drug	Target	Inhibitor type	References
**RG7155**	CSF-1R	mAb	(168)
**Pexidartinib**	CSF-1R	Small molecule	(169)
**JX-594**	GM-CSFR	Small molecule	(170)
**GVAX**	GM-CSFR	Allogeneic colon cancer cell vaccine	(171, 172)
**Durvalumab**	PD-L1	mAb	(169)

In addition, granulocyte-macrophage colony stimulating factor (GM-CSF) can enhance the function of macrophages and other immune cells and improve the antitumor and anti-infective immunity of the body (175). GM-CSF is widely used in clinical research. A clinical trial demonstrated the safety and feasibility of the GM-CSF colon cancer vaccine administered to patients with mCRC and recommended that it is necessary to further study the efficacy and antitumor immunity of this vaccine (176, 177). JX-594 is recombinant vaccinia granulosa cell-macrophage colony stimulating factor (RAC VAC GM-CSF). It has been proved that intravenous infusion of Pexa-Vec (JX-594) is a safe and well-tolerated drug (170). At present, a phase 2 study of Pexa-Vec combined with irinotecan in patients with mCRC is currently under way (178). Drugs targeting epigenomes include DNA methyltransferase 1 inhibitor (DNMTi) and histone deacetylase inhibitor (HDACi). The trial of the second generation DNMTi guadecitabine combined with colon vaccine (GVAX) secreting GM-CSF in the treatment of advanced CRC showed that the treatment was well tolerated and had no accidental toxicity, but it is closely related to the order of administration sequence (171, 172). These drugs, which have entered into clinical trials, show the potential of targeting macrophages in the treatment of CRC metastasis.

## Discussion

Clinical studies have shown that patients with mCRC have short survival time and poor prognosis, which indicate that inhibiting the metastasis of CRC is the critical point to treatment (179). With the effective application of immune checkpoint inhibitors in the treatment of melanoma, the prospects of immunotherapy for the treatment of other cancer types, including CRC, have been gradually proposed. The inhibition of immune checkpoints can enhance the tumor immune response and inhibit tumor development. PD-1 and TIM-3 are two significant immunosuppressive molecules, which have a crucial effect on immune escape and tumor development (180). PD-1 exists on the surface of T lymphocytes and is bound by its ligand PD-L1 expressed on Tregs or tumor cells, causing the reduction of tumor immunity (181–183). PD-L1 expression is a suitable prognostic biomarker to predict the survival of patients with CRC. In stage I–III CRC patients, the upregulated expression of TIM-3 and PD-1 may predict poor prognosis (180). Furthermore, there are significant differences in expression between metastatic and primary tumors. PD-1 expression in tumor-infiltrating lymphocytes is a strong prognostic indicator for CRC patients following pulmonary resection for CRC metastasis (182). In addition, lymphocyte activation gene 3 (LAG3) is also an immune checkpoint protein (184). Blocking LAG3 can enhance tumor-infiltrating T cell response in patients with mismatch-repair proficient liver metastasis of CRC (185), which might be a newfound immunotherapy target for CRC liver metastasis. CTLA-4 expressed by T cells can also inhibit the activity of CD8+ T cells and tumor immunity. All the above-mentioned proteins are important immune checkpoints, which can suppress T effector cell proliferation and consequently inhibit tumor immunity (186). Clinically, PD-1 inhibitors are effective in mCRC with mismatch repair defects and high microsatellite instability (dMMR-MSI-H), which provides a rationale for the development and application of immunotherapy in mCRC (187). As one of the most abundant immune cell types in the TIME, TAMs have fundamental significance for their development as potential targets in tumor therapy. Studies have shown that the phagocytic ability of PD-1+ TAMs is decreased, thus PD-1 inhibitors also play a critical role in the targeted therapy of TAMs (173).

In TIME, some molecules, as cellular receptors, can directly target TAMs by inhibiting the development of CRC through their inhibitors. Some molecules, such as cytokines or non-coding RNA, that activate tumor-related signal pathways can promote the immunosuppression of TIME, increase the recruitment and infiltration of immunosuppressive cells, promote EMT and tumor angiogenesis, and indirectly promote the CRC metastasis through the crosstalk between macrophages and tumor cells. Also some molecules, as cell products, can induce the polarization of M2 macrophages and predict a poor prognosis in patients.

Researchers have identified a number of factors that regulate macrophages, and we have classified and summarized their findings. According to **Table 1**, we can regulate or block the molecules that interfere with TAM infiltration or M2-like polarization, so that their depletion and reprogramming can then inhibit CRC metastasis. At present, there are few studies on direct targeting of macrophages. With the rise of nanometer technology and its application in tumor treatment (188), we hypothesize that we can use the existing nanomaterial-targeting technology to identify the unique surface markers that directly and specifically bind to TAMs and to remove or block their synergistic invasion of CRC cells; however, this technology needs to be further investigated by researchers. Inhibitors or gene knockout methods can be used in *in vivo* and *in vitro* experiments to regulate related molecules, directly or indirectly inhibit the tumor-promoting effect of TAMs, and then induce targeted macrophages to interfere with the process of CRC metastasis.

At present, there are still numerous challenges for the development of macrophages as molecular targeted therapy for tumors. Most biomarkers associated with macrophages play an indirect role, but their effects are not completely clear. Furtherly, TAM-associated molecular targets and their therapeutic effects on CRC still require verification using experimental models. Tumor treatment that relates to macrophages has entered clinical application and the associated immunotherapeutic and targeted therapy has shown the effective potential to inhibit tumor metastasis, but its clinical application is still very limited and requires further exploration before its therapeutic benefits are expanded as an intervention for tumor metastasis.

Although targeted drug therapy has achieved a certain degree of therapeutic efficacy, these agents are not effective for all patients. Besides, prolonged treatment with targeted drugs may also result in drug resistance. Studies have shown that TAMs are associated with drug resistance to bevacizumab, and TAMs secrete IL-8 which induces drug resistance to lapatinib by activating EGFR signaling (189). In a phase III clinical trial, the addition of cetuximab to mCRC patients who were treated with chemotherapy combined with bevacizumab activated M2 macrophages and reduced the progression-free survival rate (190). Thus, targeted drugs require further experimental evaluation despite their potential benefits for the treatment of cancers.

Targeted drug therapy is also limited by the degree of toxicity during treatment. Adverse effects mainly include skin toxicity and gastrointestinal reactions. Thus, the control of side effects is also a key point to be considered in the development of targeted drugs. Furthermore, exploration of therapeutic doses should also consider the maximal doses tolerated based on the condition of the patients in order to achieve the best therapeutic outcome and simultaneously to improve their quality of life during treatment. Therefore, before targeted drugs are fully applied in clinical practice, well-organized clinical trials are needed to fully elucidate the advantages of the approach and to determine ways to avoid side effects as much as possible. If these strategies can be applied to the human body after improvement, they can be used as supplementary strategies for routine treatment, which could prolong survival time and improve life quality of patients with advanced CRC.

In conclusion, CRC metastasis is a complex process associated with the interaction between tumor cells and their metastatic niche. In this paper, we described the feedback loop between CRC cells and TAMs in TIME during metastasis. As the main immune cells in the TIME, macrophages play a pivotal impact in the development of mCRC. Macrophages may exert tumoricidal effects as the M1 subtype and participate in tumor immunity. Conversely, macrophages also inhibit inflammatory reaction as in the M2 subtype and facilitate the development of mCRC. In the TIME of CRC, TAMs interact with cytokines, cell metabolites, and signaling pathways to regulate the TME of CRC. We summarized the biomolecular markers associated with macrophage activity in the mCRC TIME and provided an outline of the rationale for the development of novel molecular targeted therapy for mCRC. Accordingly, targeting TAMs is a promising strategy for CRC metastasis immunotherapy.

## Author Contributions

YinZ contributed to design the article structure, writing–review and editing, and draw the figures and tables. YiyZ contributed to writing-original draft and draw the figures and tables. QL contributed to conceptualization and funding acquisition. YW contributed to supervision, funding acquisition, and project administration. All authors contributed to the article and approved the submitted version.

## Funding

This work was supported by the National Natural Science Foundation of China (81774095, 82074232, 82030118).

## Conflict of Interest

The authors declare that the research was conducted in the absence of any commercial or financial relationships that could be construed as a potential conflict of interest.

## References

[1] Keum N Giovannucci E. Global Burden of Colorectal Cancer: Emerging Trends, Risk Factors and Prevention Strategies. Nat Rev Gastroenterol Hepatol (2019) 16(12):713–32. 10.1038/s41575-019-0189-8 31455888

[2] Silva-Fisher JM Dang HX White NM Strand MS Krasnick BA Rozycki EB. Long Non-Coding RNA RAMS11 Promotes Metastatic Colorectal Cancer Progression. Nat Commun (2020) 11(1):2156. 10.1038/s41467-020-15547-8 32358485 PMC7195452

[3] Zhang T Guo J Gu J Wang Z Wang G Li H. Identifying the Key Genes and microRNAs in Colorectal Cancer Liver Metastasis by Bioinformatics Analysis and In Vitro Experiments. Oncol Rep (2019) 41(1):279–91. 10.3892/or.2018.6840 PMC6278419 30542696

[4] Mitry E Guiu B Cosconea S Jooste V Faivre J Bouvier AM. Epidemiology, Management and Prognosis of Colorectal Cancer With Lung Metastases: A 30-Year Population-Based Study. Gut (2010) 59(10):1383–8. 10.1136/gut.2010.211557 20732912

[5] Chen R-X Chen X Xia L-P Zhang J-X Pan Z-Z Ma X-D. N-Methyladenosine Modification of Circnsun2 Facilitates Cytoplasmic Export and Stabilizes HMGA2 to Promote Colorectal Liver Metastasis. Nat Commun (2019) 10(1):4695. 10.1038/s41467-019-12651-2 31619685 PMC6795808

[6] Van Cutsem E Danielewicz I Saunders MP Pfeiffer P Argilés G Borg C. Trifluridine/tipiracil Plus Bevacizumab in Patients With Untreated Metastatic Colorectal Cancer Ineligible for Intensive Therapy: The Randomized TASCO1 Study. Ann Oncol: Off J Eur Soc Med Oncol (2020) 31(9):1160–8. 10.1016/j.annonc.2020.05.024 32497736

[7] Kopetz S Grothey A Yaeger R Van Cutsem E Desai J Yoshino T. Encorafenib, Binimetinib, and Cetuximab in V600E-Mutated Colorectal Cancer. N Engl J Med (2019) 381(17):1632–43. 10.1056/NEJMoa1908075 31566309

[8] Chen J Dai J Kang Z Yang T Zhao Q Zheng J. A Combinatorial Strategy for Overcoming Primary and Acquired Resistance of MEK Inhibition in Colorectal Cancer. Exp Cell Res (2020) 393(1):112060. 10.1016/j.yexcr.2020.112060 32407729

[9] Taghizadeh H Mader RM Müllauer L Erhart F Kautzky-Willer A Prager GW. Precision Medicine for the Management of Therapy Refractory Colorectal Cancer. J Pers Med (2020) 10(4):272. 10.3390/jpm10040272 PMC7768503 33322358

[10] Qi L Zhang Y Song F Han Y Ding Y. A Newly Identified Small Molecular Compound Acts as a Protein Kinase Inhibitor to Suppress Metastasis of Colorectal Cancer. Bioorg Chem (2021) 107:104625. 10.1016/j.bioorg.2021.104625 33454506

[11] Gao S Li N Gao S Xue Q Ying J Wang S. Neoadjuvant PD-1 Inhibitor (Sintilimab) in NSCLC. J Thorac Oncol (2020) 15(5):816–26. 10.1016/j.jtho.2020.01.017 32036071

[12] Chalabi M Fanchi LF Dijkstra KK Van den Berg JG Aalbers AG Sikorska K. Neoadjuvant Immunotherapy Leads to Pathological Responses in MMR-Proficient and MMR-Deficient Early-Stage Colon Cancers. Nat Med (2020) 26(4):566–76. 10.1038/s41591-020-0805-8 32251400

[13] Pitt JM Marabelle A Eggermont A Soria JC Kroemer G Zitvogel L. Targeting the Tumor Microenvironment: Removing Obstruction to Anticancer Immune Responses and Immunotherapy. Ann Oncol (2016) 27(8):1482–92. 10.1093/annonc/mdw168 27069014

[14] Wu J-Y Huang T-W Hsieh Y-T Wang Y-F Yen C-C Lee G-L. Cancer-Derived Succinate Promotes Macrophage Polarization and Cancer Metastasis via Succinate Receptor. Mol Cell (2020) 77(2):213–27.e5. 10.1016/j.molcel.2019.10.023 31735641

[15] Wang X Luo G Zhang K Cao J Huang C Jiang T. Hypoxic Tumor-Derived Exosomal miR-301a Mediates M2 Macrophage Polarization *via* PTEN/Pi3kγ to Promote Pancreatic Cancer Metastasis. Cancer Res (2018) 78(16):4586–98. 10.1158/0008-5472.CAN-17-3841 29880482

[16] Xia Y Rao L Yao H Wang Z Ning P Chen X. Engineering Macrophages for Cancer Immunotherapy and Drug Delivery. Adv Mater (Deerfield Beach Fla) (2020) 32(40):e2002054. 10.1158/0008-5472.CAN-17-3841 32856350

[17] Wang H Wang X Xu L Zhang J Cao H. Analysis of the Transcriptomic Features of Microsatellite Instability Subtype Colon Cancer. BMC Cancer (2019) 19(1):605. 10.1186/s12885-019-5802-2 31221124 PMC6585086

[18] van den Ende T van den Boorn HG Hoonhout NM van Etten-Jamaludin FS Meijer SL Derks S. Priming the Tumor Immune Microenvironment With Chemo(Radio)Therapy: A Systematic Review Across Tumor Types. Biochim Biophys Acta Rev Cancer (2020) 1874(1):188386. 10.1016/j.bbcan.2020.188386 32540465

[19] Huang J Li J Zheng S Lu Z Che Y Mao S. Tumor Microenvironment Characterization Identifies Two Lung Adenocarcinoma Subtypes With Specific Immune and Metabolic State. Cancer Sci (2020) 111(6):1876–86. 10.1111/cas.14390 PMC7293093 32187778

[20] Szabo PA Levitin HM Miron M Snyder ME Senda T Yuan J. Single-Cell Transcriptomics of Human T Cells Reveals Tissue and Activation Signatures in Health and Disease. Nat Commun (2019) 10(1):4706. 10.1038/s41467-019-12464-3 31624246 PMC6797728

[21] Park SM Do-Thi VA Lee J-O Lee H Kim YS. Interleukin-9 Inhibits Lung Metastasis of Melanoma Through Stimulating Anti-Tumor M1 Macrophages. Mol Cells (2020) 43(5):479–90. 10.14348/molcells.2020.0047 PMC7264476 32326670

[22] Sanchez-Correa B Valhondo I Hassouneh F Lopez-Sejas N Pera A Bergua JM. DNAM-1 and the TIGIT/PVRIG/TACTILE Axis: Novel Immune Checkpoints for Natural Killer Cell-Based Cancer Immunotherapy. Cancers (2019) 11(6):877. 10.3390/cancers11060877 PMC6628015 31234588

[23] Arlauckas SP Garren SB Garris CS Kohler RH Oh J Pittet MJ. Arg1 Expression Defines Immunosuppressive Subsets of Tumor-Associated Macrophages. Theranostics (2018) 8(21):5842–54. 10.7150/thno.26888 PMC6299430 30613266

[24] Tavazoie MF Pollack I Tanqueco R Ostendorf BN Reis BS Gonsalves FC. LXR/ApoE Activation Restricts Innate Immune Suppression in Cancer. Cell (2018) 172(4):825–40.e18. 10.1016/j.cell.2017.12.026 PMC5846344 29336888

[25] Liu C Chikina M Deshpande R Menk AV Wang T Tabib T. Treg Cells Promote the SREBP1-Dependent Metabolic Fitness of Tumor-Promoting Macrophages *via* Repression of CD8 T Cell-Derived Interferon-γ. Immunity (2019) 51(2):381–97.e6. 10.1016/j.immuni.2019.06.017 PMC6703933 31350177

[26] Derynck R Turley SJ Akhurst RJ. Tgfβ Biology in Cancer Progression and Immunotherapy. Nat Rev Clin Oncol (2021) 18(1):9–34. 10.1038/s41571-020-0403-1 32710082 PMC9721352

[27] Liu J Wu S Zheng X Zheng P Fu Y Wu C. Immune Suppressed Tumor Microenvironment by Exosomes Derived From Gastric Cancer Cells *via* Modulating Immune Functions. Sci Rep (2020) 10(1):14749. 10.1038/s41598-020-71573-y 32901082 PMC7479614

[28] Rosalia RA Quakkelaar ED Redeker A Khan S Camps M Drijfhout JW. Dendritic Cells Process Synthetic Long Peptides Better Than Whole Protein, Improving Antigen Presentation and T-Cell Activation. Eur J Immunol (2013) 43(10):2554–65. 10.1002/eji.201343324 23836147

[29] Xie Z Zheng J Wang Y Li D Maermaer T Li Y. Deficient IL-2 Produced by Activated CD56 T Cells Contributes to Impaired NK Cell-Mediated ADCC Function in Chronic HIV-1 Infection. Front Immunol (2019) 10:1647. 10.3389/fimmu.2019.01647 31379845 PMC6648879

[30] Su S Zhao J Xing Y Zhang X Liu J Ouyang Q. Immune Checkpoint Inhibition Overcomes ADCP-Induced Immunosuppression by Macrophages. Cell (2018) 175(2):442–57.e23. 10.1016/j.cell.2018.09.007 30290143

[31] Lee S-H Bar-Haim E Machlenkin A Goldberger O Volovitz I Vadai E. In Vivo Rejection of Tumor Cells Dependent on CD8 Cells That Kill Independently of Perforin and FasL. Cancer Gene Ther (2004) 11(3):237–48. 10.1038/sj.cgt.7700678 14739939

[32] Kearney CJ Vervoort SJ Hogg SJ Ramsbottom KM Freeman AJ Lalaoui N. Tumor Immune Evasion Arises Through Loss of TNF Sensitivity. Sci Immunol (2018) 3(23):eaar3451. 10.1126/sciimmunol.aar3451 29776993

[33] Beury DW Parker KH Nyandjo M Sinha P Carter KA Ostrand-Rosenberg S. Cross-Talk Among Myeloid-Derived Suppressor Cells, Macrophages, and Tumor Cells Impacts the Inflammatory Milieu of Solid Tumors. J Leukoc Biol (2014) 96(6):1109–18. 10.1189/jlb.3A0414-210R PMC4226789 25170116

[34] Huang B Pan PY Li Q Sato AI Levy DE Bromberg J. Gr-1+CD115+ Immature Myeloid Suppressor Cells Mediate the Development of Tumor-Induced T Regulatory Cells and T-Cell Anergy in Tumor-Bearing Host. Cancer Res (2006) 66(2):1123–31. 10.1158/0008-5472.CAN-05-1299 16424049

[35] Mandai M Hamanishi J Abiko K Matsumura N Baba T Konishi I. Dual Faces of IFNγ in Cancer Progression: A Role of PD-L1 Induction in the Determination of Pro- and Antitumor Immunity. Clin Cancer Res (2016) 22(10):2329–34. 10.1158/1078-0432.CCR-16-0224 27016309

[36] Syed Khaja AS Toor SM El Salhat H Ali BR Elkord E. Intratumoral FoxP3(+)Helios(+) Regulatory T Cells Upregulating Immunosuppressive Molecules Are Expanded in Human Colorectal Cancer. Front Immunol (2017) 8:619. 10.3389/fimmu.2017.00619 28603527 PMC5445103

[37] Zhang X Li W Sun J Yang Z Guan Q Wang R. How to Use Macrophages to Realise the Treatment of Tumour. J Drug Target (2020) 28(10):1034–45. 10.1080/1061186X.2020.1775236 32603199

[38] Väyrynen JP Haruki K Lau MC Väyrynen SA Zhong R Dias Costa A. The Prognostic Role of Macrophage Polarization in the Colorectal Cancer Microenvironment. Cancer Immunol Res (2021) 9(1):8–19. 10.1158/2326-6066.CIR-20-0527 33023967 PMC7785652

[39] Ma H Li Y-N Song L Liu R Li X Shang Q. Macrophages Inhibit Adipogenic Differentiation of Adipose Tissue Derived Mesenchymal Stem/Stromal Cells by Producing Pro-Inflammatory Cytokines. Cell Biosci (2020) 10:88. 10.1186/s13578-020-00450-y 32699606 PMC7372775

[40] Quero L Hanser E Manigold T Tiaden AN Kyburz D. TLR2 Stimulation Impairs Anti-Inflammatory Activity of M2-Like Macrophages, Generating a Chimeric M1/M2 Phenotype. Arthritis Res Ther (2017) 19(1):245. 10.1186/s13075-017-1447-1 29096690 PMC5667453

[41] Ma M Wang X Liu N Shan F Feng Y. Low-Dose Naltrexone Inhibits Colorectal Cancer Progression and Promotes Apoptosis by Increasing M1-Type Macrophages and Activating the Bax/Bcl-2/Caspase-3/PARP Pathway. Int Immunopharmacol (2020) 83:106388. 10.1016/j.intimp.2020.106388 32171145

[42] Sun Y Diao F Niu Y Li X Zhou H Mei Q. Apple Polysaccharide Prevents From Colitis-Associated Carcinogenesis Through Regulating Macrophage Polarization. Int J Biol Macromol (2020) 161:704–11. 10.1016/j.ijbiomac.2020.06.121 32544579

[43] Ma PF Gao CC Yi J Zhao JL Liang SQ Zhao Y. Cytotherapy With M1-Polarized Macrophages Ameliorates Liver Fibrosis by Modulating Immune Microenvironment in Mice. J Hepatol (2017) 67(4):770–9. 10.1016/j.jhep.2017.05.022 28596109

[44] Shi Y Luo P Wang W Horst K Bläsius F Relja B. M1 But Not M0 Extracellular Vesicles Induce Polarization of RAW264.7 Macrophages Via the TLR4-NFκB Pathway In Vitro. Inflammation (2020) 43(5):1611–9. 10.1007/s10753-020-01236-7 PMC7476919 32323096

[45] Zhang F Parayath NN Ene CI Stephan SB Koehne AL Coon ME. Genetic Programming of Macrophages to Perform Anti-Tumor Functions Using Targeted mRNA Nanocarriers. Nat Commun (2019) 10(1):3974. 10.1038/s41467-019-11911-5 31481662 PMC6722139

[46] Lin Y Zhao JL Zheng QJ Jiang X Tian J Liang SQ. Notch Signaling Modulates Macrophage Polarization and Phagocytosis Through Direct Suppression of Signal Regulatory Protein α Expression. Front Immunol (2018) 9:1744. 10.3389/fimmu.2018.01744 30105024 PMC6077186

[47] Rösner T Kahle S Montenegro F Matlung HL Jansen JHM Evers M. Immune Effector Functions of Human IgG2 Antibodies Against EGFR. Mol Cancer Ther (2019) 18(1):75–88. 10.1158/1535-7163.MCT-18-0341 30282813

[48] Ramesh A Kumar S Brouillard A Nandi D Kulkarni A. A Nitric Oxide (NO) Nanoreporter for Noninvasive Real-Time Imaging of Macrophage Immunotherapy. Adv Mater (2020) 32(24):e2000648. 10.1002/adma.202000648 32390270

[49] Baer C Squadrito ML Laoui D Thompson D Hansen SK Kiialainen A. Suppression of microRNA Activity Amplifies IFN-γ-Induced Macrophage Activation and Promotes Anti-Tumour Immunity. Nat Cell Biol (2016) 18(7):790–802. 10.1038/ncb3371 27295554

[50] Lee C Jeong H Bae Y Shin K Kang S Kim H. Targeting of M2-Like Tumor-Associated Macrophages With a Melittin-Based Pro-Apoptotic Peptide. J Immunother Cancer (2019) 7(1):147. 10.1186/s40425-019-0610-4 31174610 PMC6555931

[51] Oishi S Takano R Tamura S Tani S Iwaizumi M Hamaya Y. M2 Polarization of Murine Peritoneal Macrophages Induces Regulatory Cytokine Production and Suppresses T-Cell Proliferation. Immunology (2016) 149(3):320–8. 10.1111/imm.12647 PMC5046056 27421990

[52] Su B Han H Gong Y Li X Ji C Yao J. Let-7d Inhibits Intratumoral Macrophage M2 Polarization and Subsequent Tumor Angiogenesis by Targeting IL-13 and IL-10. Cancer Immunol Immunother (2020) 70(6):1619–34. 10.1007/s00262-020-02791-6 PMC10991601 33237349

[53] Dou C Ding N Zhao C Hou T Kang F Cao Z. Estrogen Deficiency-Mediated M2 Macrophage Osteoclastogenesis Contributes to M1/M2 Ratio Alteration in Ovariectomized Osteoporotic Mice. J Bone Miner Res (2018) 33(5):899–908. 10.1002/jbmr.3364 29281118

[54] Sui H Tan H Fu J Song Q Jia R Han L. The Active Fraction of Garcinia Yunnanensis Suppresses the Progression of Colorectal Carcinoma by Interfering With Tumorassociated Macrophage-Associated M2 Macrophage Polarization *In Vivo* and In Vitro. FASEB J: Off Publ Fed Am Soc Exp Biol (2020) 34(6):7387–403. 10.1096/fj.201903011R 32283574

[55] Wang Y Wei B Gao J Cai X Xu L Zhong H. Combination of Fruquintinib and Anti-PD-1 for the Treatment of Colorectal Cancer. J Immunol (2020) 205(10):2905–15. 10.4049/jimmunol.2000463 33028620

[56] Kim Y Wen X Bae JM Kim JH Cho NY Kang GH. The Distribution of Intratumoral Macrophages Correlates With Molecular Phenotypes and Impacts Prognosis in Colorectal Carcinoma. Histopathology (2018) 73(4):663–71. 10.1111/his.13674 29906313

[57] Min AKT Mimura K Nakajima S Okayama H Saito K Sakamoto W. Therapeutic Potential of Anti-VEGF Receptor 2 Therapy Targeting for M2-Tumor-Associated Macrophages in Colorectal Cancer. Cancer Immunol Immunother (2021) 70(2):289–98. 10.1007/s00262-020-02676-8 PMC10991089 32705303

[58] Ye YC Zhao JL Lu YT Gao CC Yang Y Liang SQ. NOTCH Signaling *via* WNT Regulates the Proliferation of Alternative, CCR2-Independent Tumor-Associated Macrophages in Hepatocellular Carcinoma. Cancer Res (2019) 79(16):4160–72. 10.1158/0008-5472.CAN-18-1691 31266773

[59] Zhu D Johnson TK Wang Y Thomas M Huynh K Yang Q. Macrophage M2 Polarization Induced by Exosomes From Adipose-Derived Stem Cells Contributes to the Exosomal Proangiogenic Effect on Mouse Ischemic Hindlimb. Stem Cell Res Ther (2020) 11(1):162. 10.1186/s13287-020-01669-9 32321589 PMC7178595

[60] Ding J Yang C Zhang Y Wang J Zhang S Guo D. M2 Macrophage-Derived G-CSF Promotes Trophoblasts EMT, Invasion and Migration *via* Activating PI3K/Akt/Erk1/2 Pathway to Mediate Normal Pregnancy. J Cell Mol Med (2021) 25(4):2136–47. 10.1111/jcmm.16191 PMC7882967 33393205

[61] Wang Z Du Z Sheng H Xu X Wang W Yang J. Polarization of Intestinal Tumour-Associated Macrophages Regulates the Development of Schistosomal Colorectal Cancer. J Cancer (2021) 12(4):1033–41. 10.7150/jca.48985 PMC7797650 33442402

[62] Wu MF Lin CA Yuan TH Yeh HY Su SF Guo CL. The M1/M2 Spectrum and Plasticity of Malignant Pleural Effusion-Macrophage in Advanced Lung Cancer. Cancer Immunol Immunother (2021) 70(5):1435–50. 10.1007/s00262-020-02781-8 PMC8053174 33175182

[63] Cassetta L Pollard JW. Tumor-Associated Macrophages. Curr Biol (2020) 30(6):R246–R8. 10.1007/s00262-020-02781-8 32208142

[64] Mougiakakos D Bach C Böttcher M Beier F Röhner L Stoll A. The IKZF1-IRF4/IRF5 Axis Controls Polarization of Myeloma-Associated Macrophages. Cancer Immunol Res (2021) 9(3):265–78. 10.1158/2326-6066.CIR-20-0555 33563611

[65] Nie W Wu G Zhang J Huang LL Ding J Jiang A. Responsive Exosome Nano-Bioconjugates for Synergistic Cancer Therapy. Angew Chem (Int Ed English) (2020) 59(5):2018–22. 10.1002/anie.201912524 31746532

[66] Anastas JN Moon RT. WNT Signalling Pathways as Therapeutic Targets in Cancer. Nat Rev Cancer (2013) 13(1):11–26. 10.1038/nrc3419 23258168

[67] Parker TW Neufeld KL. APC Controls Wnt-Induced Beta-Catenin Destruction Complex Recruitment in Human Colonocytes. Sci Rep (2020) 10(1):2957. 10.1038/s41598-020-59899-z 32076059 PMC7031393

[68] Soldevilla B Carretero-Puche C Gomez-Lopez G Al-Shahrour F Riesco MC Gil-Calderon B. The Correlation Between Immune Subtypes and Consensus Molecular Subtypes in Colorectal Cancer Identifies Novel Tumour Microenvironment Profiles, With Prognostic and Therapeutic Implications. Eur J Cancer (Oxf Engl 1990) (2019) 123:118–29. 10.1016/j.ejca.2019.09.008 31678770

[69] Wang B Tian T Kalland KH Ke X Qu Y. Targeting Wnt/beta-Catenin Signaling for Cancer Immunotherapy. Trends Pharmacol Sci (2018) 39(7):648–58. 10.1016/j.tips.2018.03.008 29678298

[70] Metzger R Maruskova M Krebs S Janssen KP Krug AB. Increased Incidence of Colon Tumors in AOM-Treated Apc (1638n/+) Mice Reveals Higher Frequency of Tumor Associated Neutrophils in Colon Than Small Intestine. Front Oncol (2019) 9:1001. 10.3389/fonc.2019.01001 31681563 PMC6797844

[71] Buhrmann C Shayan P Banik K Kunnumakkara AB Kubatka P Koklesova L. Targeting NF-κB Signaling by Calebin A, a Compound of Turmeric, in Multicellular Tumor Microenvironment: Potential Role of Apoptosis Induction in CRC Cells. Biomedicines (2020) 8(8):236. 10.3390/biomedicines8080236 PMC7460490 32708030

[72] Yang Y Weng W Peng J Hong L Yang L Toiyama Y. Fusobacterium Nucleatum Increases Proliferation of Colorectal Cancer Cells and Tumor Development in Mice by Activating Toll-Like Receptor 4 Signaling to Nuclear Factor-κB, and Up-Regulating Expression of MicroRNA-21. Gastroenterology (2017) 152(4):851–66.e24. 10.1053/j.gastro.2016.11.018 PMC5555435 27876571

[73] Wu Y Konaté MM Lu J Makhlouf H Chuaqui R Antony S. IL-4 and IL-17a Cooperatively Promote Hydrogen Peroxide Production, Oxidative DNA Damage, and Upregulation of Dual Oxidase 2 in Human Colon and Pancreatic Cancer Cells. J Immunol (2019) 203(9):2532–44. 10.4049/jimmunol.1800469 PMC6804760 31548328

[74] Huang M Wu R Chen L Peng Q Li S Zhang Y. S100A9 Regulates MDSCs-Mediated Immune Suppression *via* the RAGE and TLR4 Signaling Pathways in Colorectal Carcinoma. Front Immunol (2019) 10:2243. 10.3389/fimmu.2019.02243 31620141 PMC6759487

[75] Sasidharan Nair V Toor S M Taha R Z Ahmed AA Kurer MA Murshed K. Transcriptomic Profiling of Tumor-Infiltrating CD4+TIM-3+ T Cells Reveals Their Suppressive, Exhausted, and Metastatic Characteristics in Colorectal Cancer Patients. Vaccines (2020) 8(1). 10.3390/vaccines8010071 PMC7157206 32041340

[76] Yang C Shi S Su Y Tong JS Li L. P2X7R Promotes Angiogenesis and Tumour-Associated Macrophage Recruitment by Regulating the NF-κB Signalling Pathway in Colorectal Cancer Cells. J Cell Mol Med (2020) 24(18):10830–41. 10.1111/jcmm.15708 PMC7521273 32735377

[77] Zhang T Liu L Lai W Zeng Y Xu H Lan Q. Interaction With Tumor-Associated Macrophages Promotes PRL-3-induced Invasion of Colorectal Cancer Cells *via* MAPK Pathway-Induced EMT and NF-κB Signaling-Induced Angiogenesis. Oncol Rep (2019) 41(5):2790–802. 10.3892/or.2019.7049 PMC6448091 30864736

[78] Porta C Ippolito A Consonni FM Carraro L Celesti G Correale C. Protumor Steering of Cancer Inflammation by P50 NF-κB Enhances Colorectal Cancer Progression. Cancer Immunol Res (2018) 6(5):578–93. 10.1158/2326-6066.CIR-17-0036 29588321

[79] Giampazolias E Zunino B Dhayade S Bock F Cloix C Cao K. Mitochondrial Permeabilization Engages NF-κB-Dependent Anti-Tumour Activity Under Caspase Deficiency. Nat Cell Biol (2017) 19(9):1116–29. 10.1038/ncb3596 PMC5624512 28846096

[80] Johnson DE O’Keefe RA Grandis JR. Targeting the IL-6/JAK/STAT3 Signalling Axis in Cancer. Nat Rev Clin Oncol (2018) 15(4):234–48. 10.1038/nrclinonc.2018.8 PMC5858971 29405201

[81] Zhong Q Fang Y Lai Q Wang S He C Li A. CPEB3 Inhibits Epithelial-Mesenchymal Transition by Disrupting the Crosstalk Between Colorectal Cancer Cells and Tumor-Associated Macrophages *via* IL-6r/STAT3 Signaling. J Exp Clin Cancer Res: CR (2020) 39(1):132. 10.1186/s13046-020-01637-4 32653013 PMC7353816

[82] Wei C Yang C Wang S Shi D Zhang C Lin X. Crosstalk Between Cancer Cells and Tumor Associated Macrophages is Required for Mesenchymal Circulating Tumor Cell-Mediated Colorectal Cancer Metastasis. Mol Cancer (2019) 18(1):64. 10.1186/s12943-019-0976-4 30927925 PMC6441214

[83] Walens A DiMarco AV Lupo R Kroger BR Damrauer JS Alvarez JV. CCL5 Promotes Breast Cancer Recurrence Through Macrophage Recruitment in Residual Tumors. eLife (2019) 8:e43653. 10.7554/eLife.43653 30990165 PMC6478432

[84] Wang X Yang X Tsai Y Yang L Chuang KH Keng PC. IL-6 Mediates Macrophage Infiltration After Irradiation *via* Up-Regulation of CCL2/CCL5 in Non-Small Cell Lung Cancer. Radiat Res (2017) 187(1):50–9. 10.1667/RR14503.1 28054838

[85] Wang S Liang K Hu Q Li P Song J Yang Y. JAK2-Binding Long Noncoding RNA Promotes Breast Cancer Brain Metastasis. J Clin Invest (2017) 127(12):4498–515. 10.1172/JCI91553 PMC5707156 29130936

[86] Chun E Lavoie S Michaud M Gallini CA Kim J Soucy G. CCL2 Promotes Colorectal Carcinogenesis by Enhancing Polymorphonuclear Myeloid-Derived Suppressor Cell Population and Function. Cell Rep (2015) 12(2):244–57. 10.1016/j.celrep.2015.06.024 PMC4620029 26146082

[87] Janku F Yap TA Meric-Bernstam F. Targeting the PI3K Pathway in Cancer: Are We Making Headway? Nat Rev Clin Oncol (2018) 15(5):273–91. 10.1038/nrclinonc.2018.28 29508857

[88] Hoxhaj G Manning BD. The PI3K-AKT Network at the Interface of Oncogenic Signalling and Cancer Metabolism. Nat Rev Cancer (2020) 20(2):74–88. 10.1038/s41568-019-0216-7 31686003 PMC7314312

[89] Ren F Zhao Q Liu B Sun X Tang Y Huang H. Transcriptome Analysis Reveals GPNMB as a Potential Therapeutic Target for Gastric Cancer. J Cell Physiol (2020) 235(3):2738–52. 10.1002/jcp.29177 31498430

[90] Zhao R Song Y Wang Y Huang Y Li Z Cui Y. PD-1/PD-L1 Blockade Rescue Exhausted CD8+ T Cells in Gastrointestinal Stromal Tumours *via* the PI3K/Akt/mTOR Signalling Pathway. Cell Prolif (2019) 52(3):e12571. 10.1111/cpr.12571 30714229 PMC6536456

[91] Tan X Zhang Z Yao H Shen L. Tim-4 Promotes the Growth of Colorectal Cancer by Activating Angiogenesis and Recruiting Tumor-Associated Macrophages *via* the PI3K/AKT/mTOR Signaling Pathway. Cancer Lett (2018) 436:119–28. 10.1016/j.canlet.2018.08.012 30118845

[92] Wang D Wang X Si M Yang J Sun S Wu H. Exosome-Encapsulated miRNAs Contribute to CXCL12/CXCR4-Induced Liver Metastasis of Colorectal Cancer by Enhancing M2 Polarization of Macrophages. Cancer Lett (2020) 474:36–52. 10.1016/j.canlet.2020.01.005 31931030

[93] Klebanoff CA Crompton JG Leonardi AJ Yamamoto TN Chandran SS Eil RL. Inhibition of AKT Signaling Uncouples T Cell Differentiation From Expansion for Receptor-Engineered Adoptive Immunotherapy. JCI Insight (2017) 2(23):e95103. 10.1172/jci.insight.95103 PMC5752304 29212954

[94] Meng X Zhang S. MAPK Cascades in Plant Disease Resistance Signaling. Annu Rev Phytopathol (2013) 51:245–66. 10.1146/annurev-phyto-082712-102314 23663002

[95] Sun H Ou B Zhao S Liu X Song L Liu X. USP11 Promotes Growth and Metastasis of Colorectal Cancer *via* PPP1CA-Mediated Activation of ERK/MAPK Signaling Pathway. EBioMedicine (2019) 48:236–47. 10.1016/j.ebiom.2019.08.061 PMC6838424 31521612

[96] Yang MH Zhao L Wang L Ou-Yang W Hu SS Li WL. Nuclear lncRNA HOXD-AS1 Suppresses Colorectal Carcinoma Growth and Metastasis *via* Inhibiting HOXD3-Induced Integrin β3 Transcriptional Activating and MAPK/AKT Signalling. Mol Cancer (2019) 18(1):31. 10.1186/s12943-019-0955-9 30823921 PMC6397497

[97] Zhao H Xu Z Qin H Gao Z Gao L. miR-30b Regulates Migration and Invasion of Human Colorectal Cancer *via* SIX1. Biochem J (2014) 460(1):117–25. 10.1042/BJ20131535 24593661

[98] Xu H Zhang Y Peña MM Pirisi L Creek KE. Six1 Promotes Colorectal Cancer Growth and Metastasis by Stimulating Angiogenesis and Recruiting Tumor-Associated Macrophages. Carcinogenesis (2017) 38(3):281–92. 10.1093/carcin/bgw121 PMC5862328 28199476

[99] Zhang D Qiu X Li J Zheng S Li L Zhao H. TGF-β Secreted by Tumor-Associated Macrophages Promotes Proliferation and Invasion of Colorectal Cancer *via* miR-34a-VEGF Axis. Cell Cycle (2018) 17(24):2766–78. 10.1080/15384101.2018.1556064 PMC6343734 30523755

[100] Yang C Wei C Wang S Shi D Zhang C Lin X. Elevated CD163(+)/CD68(+) Ratio at Tumor Invasive Front is Closely Associated With Aggressive Phenotype and Poor Prognosis in Colorectal Cancer. Int J Biol Sci (2019) 15(5):984–98. 10.7150/ijbs.29836 PMC6535793 31182919

[101] Cai J Xia L Li J Ni S Song H Wu X. Tumor-Associated Macrophages Derived TGF-β−Induced Epithelial to Mesenchymal Transition in Colorectal Cancer Cells Through Smad2,3-4/Snail Signaling Pathway. Cancer Res Treat (2019) 51(1):252–66. 10.4143/crt.2017.613 PMC6333993 29690747

[102] Fakih M Ouyang C Wang C Tu TY Gozo MC Cho M. Immune Overdrive Signature in Colorectal Tumor Subset Predicts Poor Clinical Outcome. J Clin Invest (2019) 129(10):4464–76. 10.1172/JCI127046 PMC6763253 31524634

[103] McGeachy MJ Bak-Jensen KS Chen Y Tato CM Blumenschein W McClanahan T. TGF-Beta and IL-6 Drive the Production of IL-17 and IL-10 by T Cells and Restrain T(H)-17 Cell-Mediated Pathology. Nat Immunol (2007) 8(12):1390–7. 10.1038/ni1539 17994024

[104] Chung L Thiele Orberg E Geis AL Chan JL Fu K DeStefano Shields CE. Bacteroides Fragilis Toxin Coordinates a Pro-Carcinogenic Inflammatory Cascade *via* Targeting of Colonic Epithelial Cells. Cell Host Microbe (2018) 23(2):203–14.e5. 10.1016/j.chom.2018.01.007 29398651 PMC5954996

[105] Zhang Q Atsuta I Liu S Chen C Shi S Shi S. IL-17-Mediated M1/M2 Macrophage Alteration Contributes to Pathogenesis of Bisphosphonate-Related Osteonecrosis of the Jaws. Clin Cancer Res (2013) 19(12):3176–88. 10.1158/1078-0432.CCR-13-0042 PMC5558149 23616636

[106] Yue K Ma JL Jiang T Yue J Sun SK Shen JL. LncRNA RPPH1 Predicts Poor Prognosis and Regulates Cell Proliferation and Migration by Repressing P21 Expression in Gastric Cancer. Eur Rev Med Pharmacol Sci (2020) 24(21):11072–80. 10.26355/eurrev_202011_23593 33215423

[107] Liang Z-x Liu H-s Wang F-w Xiong L Zhou C Hu T. LncRNA RPPH1 Promotes Colorectal Cancer Metastasis by Interacting With TUBB3 and by Promoting Exosomes-Mediated Macrophage M2 Polarization. Cell Death Dis (2019) 10(11):829. 10.1038/s41419-019-2077-0 31685807 PMC6828701

[108] Zhang J Li S Zhang X Li C Zhang J Zhou W. LncRNA HLA-F-AS1 Promotes Colorectal Cancer Metastasis by Inducing PFN1 in Colorectal Cancer-Derived Extracellular Vesicles and Mediating Macrophage Polarization. Cancer Gene Ther (2021) 2021:10.1038/s41417-020-00276-3. 10.1038/s41417-020-00276-3 33531647

[109] Lan J Sun L Xu F Liu L Hu F Song D. M2 Macrophage-Derived Exosomes Promote Cell Migration and Invasion in Colon Cancer. Cancer Res (2019) 79(1):146–58. 10.1158/0008-5472.CAN-18-0014 30401711

[110] Zhao S Mi Y Guan B Zheng B Wei P Gu Y. Tumor-Derived Exosomal miR-934 Induces Macrophage M2 Polarization to Promote Liver Metastasis of Colorectal Cancer. J Hematol Oncol (2020) 13(1):156. 10.1186/s13045-020-00991-2 33213490 PMC7678301

[111] Akimoto M Maruyama R Takamaru H Ochiya T Takenaga K. Soluble IL-33 Receptor Sst2 Inhibits Colorectal Cancer Malignant Growth by Modifying the Tumour Microenvironment. Nat Commun (2016) 7:13589. 10.1038/ncomms13589 27882929 PMC5123057

[112] Liu Q Song J Pan Y Shi D Yang C Wang S. Wnt5a/CaMKII/ERK/CCL2 Axis Is Required for Tumor-Associated Macrophages to Promote Colorectal Cancer Progression. Int J Biol Sci (2020) 16(6):1023–34. 10.7150/ijbs.40535 PMC7053330 32140070

[113] Liu C Yao Z Wang J Zhang W Yang Y Zhang Y. Macrophage-Derived CCL5 Facilitates Immune Escape of Colorectal Cancer Cells *via* the P65/STAT3-CSN5-PD-L1 Pathway. Cell Death Differ (2020) 27(6):1765–81. 10.1038/s41418-019-0460-0 PMC7244707 31802034

[114] Wang D Sun H Wei J Cen B DuBois RN. CXCL1 Is Critical for Premetastatic Niche Formation and Metastasis in Colorectal Cancer. Cancer Res (2017) 77(13):3655–65. 10.1158/0008-5472.CAN-16-3199 PMC5877403 28455419

[115] Deng Q Geng Y Zhao L Li R Zhang Z Li K. NLRP3 Inflammasomes in Macrophages Drive Colorectal Cancer Metastasis to the Liver. Cancer Lett (2019) 442:21–30. 10.1016/j.canlet.2018.10.030 30392787

[116] Ohashi K Wang Z Yang YM Billet S Tu W Pimienta M. NOD-Like Receptor C4 Inflammasome Regulates the Growth of Colon Cancer Liver Metastasis in NAFLD. Hepatology (2019) 70(5):1582–99. 10.1002/hep.30693 PMC6819206 31044438

[117] Liu Q Yang C Wang S Shi D Wei C Song J. Wnt5a-Induced M2 Polarization of Tumor-Associated Macrophages *via* IL-10 Promotes Colorectal Cancer Progression. Cell Commun Signal (2020) 18(1):51. 10.1186/s12964-020-00557-2 32228612 PMC7106599

[118] Zang Y Dong Q Lu Y Dong K Wang R Liang Z. Lumican Inhibits Immune Escape and Carcinogenic Pathways in Colorectal Adenocarcinoma. Aging (Albany NY) (2021) 13(3):4388–408. 10.18632/aging.202401 PMC7906189 33493133

[119] Shang S Ji X Zhang L Chen J Li C Shi R. Macrophage ABHD5 Suppresses NFκB-Dependent Matrix Metalloproteinase Expression and Cancer Metastasis. Cancer Res (2019) 79(21):5513–26. 10.1158/0008-5472.CAN-19-1059 31439546

[120] Alonso A Sasin J Bottini N Friedberg I Friedberg I Osterman A. Protein Tyrosine Phosphatases in the Human Genome. Cell (2004) 117(6):699–711. 10.1016/j.cell.2004.05.018 15186772

[121] Lan Q Lai W Zeng Y Liu L Li S Jin S. CCL26 Participates in the PRL-3-Induced Promotion of Colorectal Cancer Invasion by Stimulating Tumor-Associated Macrophage Infiltration. Mol Cancer Ther (2018) 17(1):276–89. 10.1158/1535-7163.MCT-17-0507 29051319

[122] Ruess DA Heynen GJ Ciecielski KJ Ai J Berninger A Kabacaoglu D. Mutant KRAS-Driven Cancers Depend on PTPN11/SHP2 Phosphatase. Nat Med (2018) 24(7):954–60. 10.1038/s41591-018-0024-8 29808009

[123] Li R Zhou R Wang H Li W Pan M Yao X. Gut Microbiota-Stimulated Cathepsin K Secretion Mediates TLR4-Dependent M2 Macrophage Polarization and Promotes Tumor Metastasis in Colorectal Cancer. Cell Death Differ (2019) 26(11):2447–63. 10.1038/s41418-019-0312-y PMC6889446 30850734

[124] Zha H Sun H Li X Duan L Li A Gu Y. S100A8 Facilitates the Migration of Colorectal Cancer Cells Through Regulating Macrophages in the Inflammatory Microenvironment. Oncol Rep (2016) 36(1):279–90. 10.3892/or.2016.4790 27176480

[125] Ayiomamitis GD Notas G Vasilakaki T Tsavari A Vederaki S Theodosopoulos T. Understanding the Interplay Between COX-2 and hTERT in Colorectal Cancer Using a Multi-Omics Analysis. Cancers (Basel) (2019) 11(10):1536. 10.3390/cancers11101536 PMC6827032 31614548

[126] Qian F Xiao J Hu B Sun N Yin W Zhu J. High Expression of P2X7R is an Independent Postoperative Indicator of Poor Prognosis in Colorectal Cancer. Hum Pathol (2017) 64:61–8. 10.1016/j.humpath.2017.03.019 28412208

[127] Pan J-H Zhou H Cooper L Huang J-L Zhu S-B Zhao X-X. LAYN Is a Prognostic Biomarker and Correlated With Immune Infiltrates in Gastric and Colon Cancers. Front Immunol (2019) 10:6. 10.3389/fimmu.2019.00006 30761122 PMC6362421

[128] Lu W Yu W He J Liu W Yang J Lin X. Reprogramming Immunosuppressive Myeloid Cells Facilitates Immunotherapy for Colorectal Cancer. EMBO Mol Med (2021) 13(1):e12798. 10.15252/emmm.202012798 33283987 PMC7799360

[129] Bellamkonda K Chandrashekar NK Osman J Selvanesan BC Savari S Sjölander A. The Eicosanoids Leukotriene D4 and Prostaglandin E2 Promote the Tumorigenicity of Colon Cancer-Initiating Cells in a Xenograft Mouse Model. BMC Cancer (2016) 16:425. 10.1186/s12885-016-2466-z 27388564 PMC4937611

[130] Campesato LF Budhu S Tchaicha J Weng CH Gigoux M Cohen IJ. Blockade of the AHR Restricts a Treg-Macrophage Suppressive Axis Induced by L-Kynurenine. Nat Commun (2020) 11(1):4011. 10.1038/s41467-020-17750-z 32782249 PMC7419300

[131] Yang C Dou R Wei C Liu K Shi D Zhang C. Tumor-Derived Exosomal microRNA-106b-5p Activates EMT-Cancer Cell and M2-Subtype TAM Interaction to Facilitate CRC Metastasis. Mol Ther (2021). 10.1016/j.ymthe.2021.02.006 PMC8178444 33571679

[132] Liu M Sun T Li N Peng J Fu D Li W. BRG1 Attenuates Colonic Inflammation and Tumorigenesis Through Autophagy-Dependent Oxidative Stress Sequestration. Nat Commun (2019) 10(1):4614. 10.1038/s41467-019-12573-z 31601814 PMC6787222

[133] Metzger R Maruskova M Krebs S Janssen K-P Krug AB. Increased Incidence of Colon Tumors in AOM-Treated Mice Reveals Higher Frequency of Tumor Associated Neutrophils in Colon Than Small Intestine. Front Oncol (2019) 9:1001. 10.3389/fonc.2019.01001 31681563 PMC6797844

[134] Liu C Lu H Wang H Loo A Zhang X Yang G. Combinations With Allosteric SHP2 Inhibitor TNO155 to Block Receptor Tyrosine Kinase Signaling. Clin Cancer Res (2021) 27(1):342–54. 10.1158/1078-0432.CCR-20-2718 33046519

[135] Nam SH Kim D Lee D Lee H-M Song D-G Jung JW. Lysyl-tRNA Synthetase-Expressing Colon Spheroids Induce M2 Macrophage Polarization to Promote Metastasis. J Clin Invest (2018) 128(11):5034–55. 10.1172/JCI99806 PMC6205414 30188867

[136] Piao C Zhang W-M Li T-T Zhang C-C Qiu S Liu Y. Complement 5a Stimulates Macrophage Polarization and Contributes to Tumor Metastases of Colon Cancer. Exp Cell Res (2018) 366(2):127–38. 10.1016/j.yexcr.2018.03.009 29551360

[137] Zhang L Li Z Ding G La X Yang P Li Z. GRP78 Plays an Integral Role in Tumor Cell Inflammation-Related Migration Induced by M2 Macrophages. Cell Signal (2017) 37:136–48. 10.1016/j.cellsig.2017.06.008 28629783

[138] Herrero A Benedicto A Romayor I Olaso E Arteta B. Inhibition of COX-2 Impairs Colon Cancer Liver Metastasis Through Reduced Stromal Cell Reaction. Biomol Ther (Seoul) (2021) 29(3):342–51. 10.4062/biomolther.2020.160 PMC8094073 33455946

[139] Sharma RA Gescher A Plastaras JP Leuratti C Singh R Gallacher-Horley B. Cyclooxygenase-2, Malondialdehyde and Pyrimidopurinone Adducts of Deoxyguanosine in Human Colon Cells. Carcinogenesis (2001) 22(9):1557–60. 10.1093/carcin/22.9.1557 11532880

[140] Huang GL Tao A Miyazaki T Khan T Hong T Nakagawa Y. PEG-Poly(1-Methyl-L-Tryptophan)-Based Polymeric Micelles as Enzymatically Activated Inhibitors of Indoleamine 2,3-Dioxygenase. Nanomater (Basel) (2019) 9(5):719. 10.3390/nano9050719 PMC6566635 31075929

[141] Wu D Zhu Y. Role of Kynurenine in Promoting the Generation of Exhausted CD8+ T Cells in Colorectal Cancer. Am J Transl Res (2021) 13(3):1535–47. PMC8014392 33841677

[142] Huang Q Xia J Wang L Wang X Ma X Deng Q. miR-153 Suppresses IDO1 Expression and Enhances CAR T Cell Immunotherapy. J Hematol Oncol (2018) 11(1):58. 10.1186/s13045-018-0600-x 29685162 PMC5914051

[143] Damato A Iachetta F Antonuzzo L Nasti G Bergamo F Bordonaro R. Phase II Study on First-Line Treatment of NIVolumab in Combination With Folfoxiri/Bevacizumab in Patients With Advanced COloRectal Cancer RAS or BRAF Mutated - NIVACOR Trial (GOIRC-03-2018). BMC Cancer (2020) 20(1):822. 10.1186/s12885-020-07268-4 32867715 PMC7457535

[144] Osumi H Shinozaki E Ooki A Wakatsuki T Kamiimabeppu D Sato T. Early Hypertension and Neutropenia are Predictors of Treatment Efficacy in Metastatic Colorectal Cancer Patients Administered FOLFIRI and Vascular Endothelial Growth Factor Inhibitors as Second-Line Chemotherapy. Cancer Med (2021) 10(2):615–25. 10.1002/cam4.3638 PMC7877370 33347731

[145] Rahma OE Hodi FS. The Intersection Between Tumor Angiogenesis and Immune Suppression. Clin Cancer Res (2019) 25(18):5449–57. 10.1158/1078-0432.CCR-18-1543 30944124

[146] Tsao LC Crosby EJ Trotter TN Agarwal P Hwang BJ Acharya C. CD47 Blockade Augmentation of Trastuzumab Antitumor Efficacy Dependent on Antibody-Dependent Cellular Phagocytosis. JCI Insight (2019) 4(24):e131882. 10.1172/jci.insight.131882 PMC6975273 31689243

[147] Goldberg SB Redman MW Lilenbaum R Politi K Stinchcombe TE Horn L. Randomized Trial of Afatinib Plus Cetuximab Versus Afatinib Alone for First-Line Treatment of EGFR-Mutant Non-Small-Cell Lung Cancer: Final Results From SWOG S1403. J Clin Oncol: Off J Am Soc Clin Oncol (2020) 38(34):4076–85. 10.1200/JCO.20.01149 PMC7768342 33021871

[148] Taniguchi H Yamanaka T Sakai D Muro K Yamazaki K Nakata S. Efficacy of Panitumumab and Cetuximab in Patients With Colorectal Cancer Previously Treated With Bevacizumab; a Combined Analysis of Individual Patient Data From ASPECCT and WJOG6510G. Cancers (Basel) (2020) 12(7):1715. 10.3390/cancers12071715 PMC7407286 32605298

[149] Zhao Y Liu X Huo M Wang Y Li Y Xu N. Cetuximab Enhances the Anti-Tumor Function of Macrophages in an IL-6 Dependent Manner. Life Sci (2021) 267:118953. 10.1016/j.lfs.2020.118953 33359746

[150] Sartore-Bianchi A Trusolino L Martino C Bencardino K Lonardi S Bergamo F. Dual-Targeted Therapy With Trastuzumab and Lapatinib in Treatment-Refractory, KRAS Codon 12/13 Wild-Type, HER2-Positive Metastatic Colorectal Cancer (HERACLES): A Proof-of-Concept, Multicentre, Open-Label, Phase 2 Trial. Lancet Oncol (2016) 17(6):738–46. 10.1016/S1470-2045(16)00150-9 27108243

[151] Hannesdóttir L Tymoszuk P Parajuli N Wasmer MH Philipp S Daschil N. Lapatinib and Doxorubicin Enhance the Stat1-Dependent Antitumor Immune Response. Eur J Immunol (2013) 43(10):2718–29. 10.1002/eji.201242505 23843024

[152] Van Cutsem E Martinelli E Cascinu S Sobrero A Banzi M Seitz JF. Regorafenib for Patients With Metastatic Colorectal Cancer Who Progressed After Standard Therapy: Results of the Large, Single-Arm, Open-Label Phase IIIb CONSIGN Study. Oncol (2019) 24(2):185–92. 10.1634/theoncologist.2018-0072 PMC6369948 30190299

[153] Li J Qin S Xu R Yau TC Ma B Pan H. Regorafenib Plus Best Supportive Care Versus Placebo Plus Best Supportive Care in Asian Patients With Previously Treated Metastatic Colorectal Cancer (CONCUR): A Randomised, Double-Blind, Placebo-Controlled, Phase 3 Trial. Lancet Oncol (2015) 16(6):619–29. 10.1016/S1470-2045(15)70156-7 25981818

[154] Ou DL Chen CW Hsu CL Chung CH Feng ZR Lee BS. Regorafenib Enhances Antitumor Immunity via Inhibition of P38 Kinase/Creb1/Klf4 Axis in Tumor-Associated Macrophages. J Immunother Cancer (2021) 9(3):e001657. 10.1136/jitc-2020-001657 33753566 PMC7986673

[155] Kim ST Hong JY Lee J Park JO Lim HY Kang WK. Pemetrexed/Erlotinib as a Salvage Treatment in Patients With High EGFR-Expressing Metastatic Colorectal Cancer Following Failure of Standard Chemotherapy: A Phase II Single-Arm Prospective Study. Target Oncol (2020) 15(1):67–73. 10.1007/s11523-019-00691-z 31820199

[156] Grépin R Guyot M Dumond A Durivault J Ambrosetti D Roussel JF. The Combination of Bevacizumab/Avastin and Erlotinib/Tarceva Is Relevant for the Treatment of Metastatic Renal Cell Carcinoma: The Role of a Synonymous Mutation of the EGFR Receptor. Theranostics (2020) 10(3):1107–21. 10.7150/thno.38346 PMC6956821 31938054

[157] Shih CT Shiau CW Chen YL Chen LJ Chao TI Wang CY. TD-92, a Novel Erlotinib Derivative, Depletes Tumor-Associated Macrophages in Non-Small Cell Lung Cancer *via* Down-Regulation of CSF-1R and Enhances the Anti-Tumor Effects of Anti-PD-1. Cancer Lett (2021) 498:142–51. 10.1016/j.canlet.2020.10.043 33232786

[158] Jonker DJ Nott L Yoshino T Gill S Shapiro J Ohtsu A. Napabucasin Versus Placebo in Refractory Advanced Colorectal Cancer: A Randomised Phase 3 Trial. Lancet Gastroenterol Hepatol (2018) 3(4):263–70. 10.1016/S2468-1253(18)30009-8 29397354

[159] Yin Y Yao S Hu Y Feng Y Li M Bian Z. The Immune-Microenvironment Confers Chemoresistance of Colorectal Cancer Through Macrophage-Derived Il6. Clin Cancer Res (2017) 23(23):7375–87. 10.1158/1078-0432.CCR-17-1283 28928161

[160] Dienstmann R Patnaik A Garcia-Carbonero R Cervantes A Benavent M Roselló S. Safety and Activity of the First-In-Class Sym004 Anti-EGFR Antibody Mixture in Patients With Refractory Colorectal Cancer. Cancer Discovery (2015) 5(6):598–609. 10.1158/2159-8290.CD-14-1432 25962717

[161] Macarulla T Cervantes A Tabernero J Roselló S Van Cutsem E Tejpar S. Phase I Study of FOLFIRI Plus Pimasertib as Second-Line Treatment for KRAS-Mutated Metastatic Colorectal Cancer. Br J Cancer (2015) 112(12):1874–81. 10.1038/bjc.2015.144 PMC4580393 25989270

[162] Fane ME Ecker BL Kaur A Marino GE Alicea GM Douglass SM. Sfrp2 Supersedes VEGF as an Age-Related Driver of Angiogenesis in Melanoma, Affecting Response to Anti-VEGF Therapy in Older Patients. Clin Cancer Res (2020) 26(21):5709–19. 10.1158/1078-0432.CCR-20-0446 PMC7642114 33097493

[163] Tabernero J Hozak RR Yoshino T Cohn AL Obermannova R Bodoky G. Analysis of Angiogenesis Biomarkers for Ramucirumab Efficacy in Patients With Metastatic Colorectal Cancer From RAISE, a Global, Randomized, Double-Blind, Phase III Study. Ann Oncol (2018) 29(3):602–9. 10.1093/annonc/mdx767 PMC5888948 29228087

[164] Liu X Lukowski JK Flinders C Kim S Georgiadis RA Mumenthaler SM. MALDI-MSI of Immunotherapy: Mapping the EGFR-Targeting Antibody Cetuximab in 3D Colon-Cancer Cell Cultures. Anal Chem (2018) 90(24):14156–64. 10.1021/acs.analchem.8b02151 PMC7250007 30479121

[165] Lai YS Wahyuningtyas R Aui SP Chang KT. Autocrine VEGF Signalling on M2 Macrophages Regulates PD-L1 Expression for Immunomodulation of T Cells. J Cell Mol Med (2019) 23(2):1257–67. 10.1111/jcmm.14027 PMC6349155 30456891

[166] Hardbower DM Coburn LA Asim M Singh K Sierra JC Barry DP. EGFR-Mediated Macrophage Activation Promotes Colitis-Associated Tumorigenesis. Oncogene (2017) 36(27):3807–19. 10.1038/onc.2017.23 PMC5501754 28263971

[167] Tamura R Tanaka T Morimoto Y Kuranari Y Yamamoto Y Takei J. Alterations of the Tumor Microenvironment in Glioblastoma Following Radiation and Temozolomide With or Without Bevacizumab. Ann Transl Med (2020) 8(6):297. 10.21037/atm.2020.03.11 32355741 PMC7186631

[168] Ries CH Cannarile MA Hoves S Benz J Wartha K Runza V. Targeting Tumor-Associated Macrophages With Anti-CSF-1R Antibody Reveals a Strategy for Cancer Therapy. Cancer Cell (2014) 25(6):846–59. 10.1016/j.ccr.2014.05.016 24898549

[169] Available at: https://clinicaltrials.gov/ct2/show/study/NCT02777710?term=macrophages&cond=Colorectal+Cancer+Metastatic&draw=2&rank=7.

[170] Park SH Breitbach CJ Lee J Park JO Lim HY Kang WK. Phase 1b Trial of Biweekly Intravenous Pexa-Vec (JX-594), an Oncolytic and Immunotherapeutic Vaccinia Virus in Colorectal Cancer. Mol Ther (2015) 23(9):1532–40. 10.1038/mt.2015.109 PMC4817877 26073886

[171] Available at: https://clinicaltrials.gov/ct2/show/NCT01966289?term=macrophages&cond=Colorectal+Cancer+Metastatic&draw=2&rank=3.

[172] Bever KM Thomas DL 2nd Zhang J Diaz Rivera EA Rosner GL Zhu Q. A Feasibility Study of Combined Epigenetic and Vaccine Therapy in Advanced Colorectal Cancer With Pharmacodynamic Endpoint. Clin Epigenet (2021) 13(1):25. 10.1186/s13148-021-01014-8 PMC7856736 33531075

[173] Gordon SR Maute RL Dulken BW Hutter G George BM McCracken MN. PD-1 Expression by Tumour-Associated Macrophages Inhibits Phagocytosis and Tumour Immunity. Nature (2017) 545(7655):495–9. 10.1038/nature22396 PMC5931375 28514441

[174] Lin EY Li JF Gnatovskiy L Deng Y Zhu L Grzesik DA. Macrophages Regulate the Angiogenic Switch in a Mouse Model of Breast Cancer. Cancer Res (2006) 66(23):11238–46. 10.1158/0008-5472.CAN-06-1278 17114237

[175] Shi Y Liu CH Roberts AI Das J Xu G Ren G. Granulocyte-Macrophage Colony-Stimulating Factor (GM-CSF) and T-Cell Responses: What We do and Don’t Know. Cell Res (2006) 16(2):126–33. 10.1038/sj.cr.7310017 16474424

[176] Available at: https://clinicaltrials.gov/ct2/show/NCT00656123?term=macrophages&cond=Colorectal+Cancer+Metastatic&draw=2&rank=1.

[177] Zheng L Edil BH Soares KC El-Shami K Uram JN Judkins C. A Safety and Feasibility Study of an Allogeneic Colon Cancer Cell Vaccine Administered With a Granulocyte-Macrophage Colony Stimulating Factor-Producing Bystander Cell Line in Patients With Metastatic Colorectal Cancer. Ann Surg Oncol (2014) 21(12):3931–7. 10.1245/s10434-014-3844-x PMC4192092 24943235

[178] Available at: https://clinicaltrials.gov/ct2/show/study/NCT01394939?term=macrophages&cond=Colorectal+Cancer+Metastatic&draw=2&rank=10.

[179] Elez E Chianese C Sanz-García E Martinelli E Noguerido A Mancuso FM. Impact of Circulating Tumor DNA Mutant Allele Fraction on Prognosis in RAS-Mutant Metastatic Colorectal Cancer. Mol Oncol (2019) 13(9):1827–35. 10.1002/1878-0261.12547 PMC6717744 31322322

[180] Kuai W Xu X Yan J Zhao W Li Y Wang B. Prognostic Impact of PD-1 and Tim-3 Expression in Tumor Tissue in Stage I-III Colorectal Cancer. BioMed Res Int (2020) 2020:5294043. 10.1155/2020/5294043 32509862 PMC7244975

[181] Yi M Jiao D Xu H Liu Q Zhao W Han X. Biomarkers for Predicting Efficacy of PD-1/PD-L1 Inhibitors. Mol Cancer (2018) 17(1):129. 10.1186/s12943-018-0864-3 30139382 PMC6107958

[182] Kollmann D Schweiger T Schwarz S Ignatova D Chang YT Lewik G. PD1-Positive Tumor-Infiltrating Lymphocytes Are Associated With Poor Clinical Outcome After Pulmonary Metastasectomy for Colorectal Cancer. Oncoimmunology (2017) 6(9):e1331194. 10.1080/2162402X.2017.1331194 28932634 PMC5599080

[183] Langhans B Nischalke HD Krämer B Dold L Lutz P Mohr R. Role of Regulatory T Cells and Checkpoint Inhibition in Hepatocellular Carcinoma. Cancer Immunol Immunother (2019) 68(12):2055–66. 10.1007/s00262-019-02427-4 PMC11028391 31724091

[184] Andrews LP Marciscano AE Drake CG Vignali DAA. LAG3 (CD223) as a Cancer Immunotherapy Target. Immunol Rev (2017) 276(1):80–96. 10.1111/imr.12519 28258692 PMC5338468

[185] Zhou G Noordam L Sprengers D Doukas M Boor PPC van Beek AA. Blockade of LAG3 Enhances Responses of Tumor-Infiltrating T Cells in Mismatch Repair-Proficient Liver Metastases of Colorectal Cancer. Oncoimmunology (2018) 7(7):e1448332. 10.1080/2162402X.2018.1448332 29900067 PMC5993483

[186] Fu X Luo H Zheng Y Wang S Zhong Z Wang Y. CTLA-4 Immunotherapy Exposes Differences in Immune Response Along With Different Tumor Progression in Colorectal Cancer. Aging (Albany NY) (2020) 12(15):15656–69. 10.18632/aging.103765 PMC7467381 32805718

[187] Overman MJ McDermott R Leach JL Lonardi S Lenz HJ Morse MA. Nivolumab in Patients With Metastatic DNA Mismatch Repair-Deficient or Microsatellite Instability-High Colorectal Cancer (CheckMate 142): An Open-Label, Multicentre, Phase 2 Study. Lancet Oncol (2017) 18(9):1182–91. 10.1016/S1470-2045(17)30422-9 PMC6207072 28734759

[188] Zhang Y Li C Jia R Gao R Zhao Y Ji Q. PEG-Poly(Amino Acid)s/EpCAM Aptamer Multifunctional Nanoparticles Arrest the Growth and Metastasis of Colorectal Cancer. Biomater Sci (2021) 9(10):3705–17. 10.1039/d1bm00160d 34008621

[189] Ahmed S Mohamed HT El-Husseiny N El Mahdy MM Safwat G Diab AA. IL-8 Secreted by Tumor Associated Macrophages Contribute to Lapatinib Resistance in HER2-Positive Locally Advanced Breast Cancer *via* Activation of Src/STAT3/ERK1/2-Mediated EGFR Signaling. Biochim Biophys Acta Mol Cell Res (2021) 1868(6):118995. 10.1016/j.bbamcr.2021.118995 33667527

[190] Pander J Heusinkveld M van der Straaten T Jordanova ES Baak-Pablo R Gelderblom H. Activation of Tumor-Promoting Type 2 Macrophages by EGFR-Targeting Antibody Cetuximab. Clin Cancer Res (2011) 17(17):5668–73. 10.1158/1078-0432.CCR-11-0239 21788356

